# Dual‐Channel Interdigitated Aptamer‐Based Sensors for Rapid Small‐Molecule Detection in Biofluids

**DOI:** 10.1002/anie.8361141

**Published:** 2026-05-14

**Authors:** Senyao Wang, Ali Elmorsy, Defne Tüzün, Lilly Schmidt, Sebastian Freko, Lukas Hiendlmeier, Chen Wang, Alonso Ingar Romero, George Al Boustani, Hu Peng, Berna Özkale, Nako Nakatsuka, Bernhard Wolfrum

**Affiliations:** ^1^ Neuroelectronics, Munich Institute of Biomedical Engineering Department of Electrical Engineering TUM School of Computation, Information and Technology Technical University of Munich Garching Germany; ^2^ Laboratory of Chemical Nanotechnology (CHEMINA) École Polytechnique Fédérale de Lausanne (EPFL) Geneva Switzerland; ^3^ TUM Center for Organoid Systems and Tissue Engineering Technical University of Munich Garching Germany; ^4^ Microrobotic Bioengineering Lab Department of Electrical Engineering TUM School of Computation, Information and Technology Technical University of Munich Garching Germany

**Keywords:** biofluid analysis, dopamine, cortisol, DNA aptamer, point‐of‐care diagnostics

## Abstract

Timely and decentralized quantification of small‐molecule biomarkers is essential for point‐of‐care (POC) diagnostics, but their reliable detection in biofluids remains challenging. Here, we introduce an engineered electrochemical aptamer sensing platform that employs a dual‐channel signal conversion strategy to overcome these limitations. By integrating spatially separated interdigitated working electrodes with selective self‐assembled monolayer (SAM) removal, the system enables target‐induced release and recapture of methylene blue‐labeled complementary DNA (MB‐cDNA) probes across two electrodes. This configuration minimizes background interference and enhances mass transport, facilitating robust dual‐channel signal transduction. The platform enables rapid (≤ 30 min), low‐volume (30 µL) detection of prototypical small molecules, dopamine and cortisol, in diverse biofluids, including artificial cerebrospinal fluid (aCSF), human serum, and saliva. Notably, the signal conversion mechanism remained effective across different targets and sample types, requiring only minimal adaptation of the recognition sequence. Together, these features establish a versatile electrochemical sensing architecture with broad potential for rapid, quantitative small‐molecule analysis in complex biological media.

## Graphical abstract

An electrochemical aptamer sensing chip employs dual interdigitated electrodes and selective self‐assembled monolayer removal to convert small‐molecule recognition into robust “signal‐off/on” responses. This dual‐channel signal conversion minimizes background noise and accelerates probe diffusion, enabling rapid (as fast as 5–30 min), low‐volume detection of dopamine and cortisol in artificial cerebrospinal fluid, serum, and saliva. The versatile architecture is readily adaptable to diverse small‐molecule targets in complex biofluids.

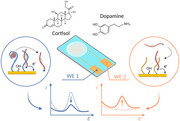

## 1 Introduction

Small‐molecule biomarkers, including neurotransmitters, hormones, and metabolites, play an indispensable role in regulating physiological processes and are intimately linked to numerous disease states [1, 2]. Timely and decentralized quantification of these targets is therefore critical for personalized medicine and POC health monitoring [3]. However, compared to macromolecular biomarkers such as proteins, small molecules pose unique analytical challenges: typically low physiological concentrations, high structural similarity to endogenous interferents, and limited epitope availability, all of which hinder both selective recognition and efficient signal transduction [4, 5]. Currently, POC detection of small molecules mostly relies on immunoassay‐based strategies, including lateral flow assays [6, 7], enzyme‐linked immunosorbent assays (ELISA) [8], and electrochemical immunosensors [9]. While these methods enable rapid analysis, each comes with inherent limitations. Lateral flow assays, though inexpensive and easy to use, often lack sensitivity and provide only qualitative or semi‐quantitative results [10]. ELISA and electrochemical immunosensors provide higher sensitivity but require multiple handling steps such as labeling, washing, and signal amplification that complicate operation and prolong the overall assay time [11]. These drawbacks limit their practicality for rapid, low‐cost testing in decentralized or resource‐limited settings.

Aptamer‐based electrochemical biosensors have emerged as a promising alternative for POC small‐molecule detection, offering the advantages of tunability of sensing parameters, minimal batch‐to‐batch variations, and compatibility with miniaturized transducers [12, 13, 14]. However, when deployed in complex biological fluids such as serum, plasma, or saliva, these systems face challenges, including biofouling‐induced signal loss [15, 16] and non‐specific protein adsorption that compromises sensing fidelity [5]. To address these issues, recent advances have explored antifouling surface coatings [17, 18, 19] to protect the electrochemical interface. Additionally, aptamers were confined within electrode nanotopographies [20, 21] or solid‐state nanopores to improve sensor longevity and performance [22, 23]. Yet, these solutions are not without trade‐offs: antifouling coatings can interfere with aptamer folding or target recognition and increase batch‐to‐batch variability, while nanoscale confinement renders the system diffusion limited, compromising temporal resolution. Thus, robust and generalizable electrochemical platforms for direct small‐molecule detection in real‐world biofluids remain elusive.

To address the aforementioned limitations, we revisited commonly employed aptamer‐based transduction strategies in electrochemical biosensors: target‐induced conformational switching and competitive displacement of labeled single‐stranded DNA [24, 25]. While both approaches are effective in principle, they often suffer from high background signals and signal loss in complex biological environments [15, 26]. We reasoned that introducing a dual‐electrode readout configuration, with one electrode serving as an internal reference, could compensate for these signal losses and improve sensing reliability. Earlier dual‐channel electrochemical systems have focused on macromolecular targets such as proteins, viruses, or bacterial markers, typically using individual separated electrodes with relatively large inter‐electrode spacing (300–500 µm), which limited probe transport and led to slow (over 1 h), diffusion‐driven readout [27, 28]. In contrast, the specific detection of small molecules with limited mass and charge presents a fundamentally different challenge, necessitating distinct design considerations in probe architecture, surface chemistry, and signal transduction. Small‐molecule binding events induce subtler recognition‐associated changes, making reliable signal conversion in complex biofluids particularly challenging.

**FIGURE 1:**
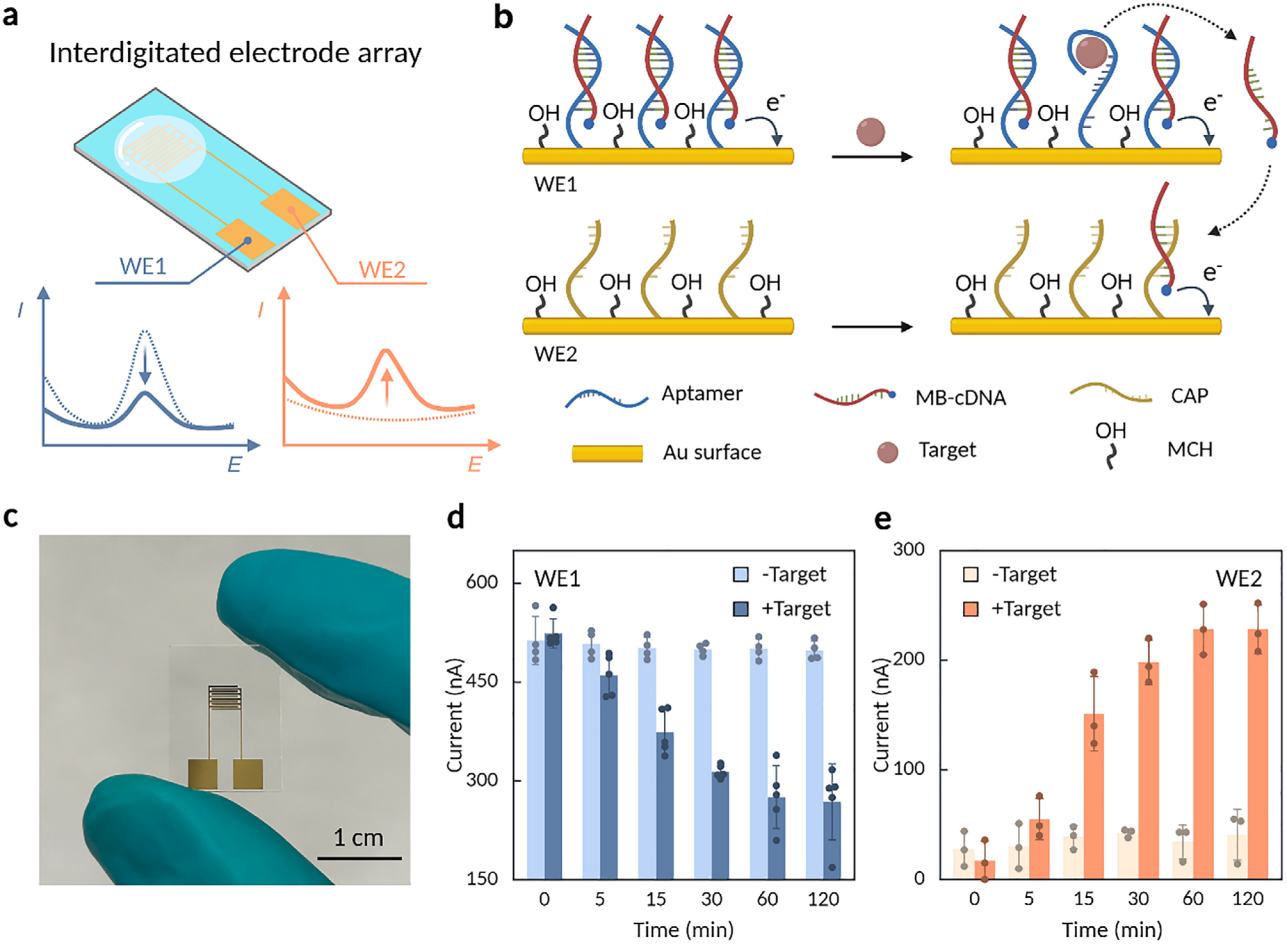
Electrochemical dual‐channel aptamer sensing platform for small‐molecule detection on an interdigitated electrode (IDE) array chip. (a) Schematic illustration of the sensor platform featuring two interdigitated working electrodes (WE1 and WE2), designed to yield opposite electrochemical responses upon target recognition. (b) Sensing mechanism: WE1 functions in a signal‐off mode, where target binding triggers the release of methylene blue‐labeled complementary DNA (MB‐cDNA). The released MB‐cDNA is subsequently recaptured by an immobilized capture probe (CAP) on WE2, generating a signal‐on response. The 6‐mercapto‐1‐hexanol (MCH) serves as the backfill molecule to optimize the density of surface‐tethered DNA. (c) Photograph of the IDE chip on a glass substrate. (d, e) Time‐dependent current responses from WE1 (d) and WE2 (e) in the absence (−Target, buffer only) and presence (+Target, 100 nM dopamine) of analyte in artificial cerebrospinal fluid (aCSF). Current data were measured by square wave voltammetry, with peak values extracted using PSTrace software. Data are presented as mean ± SD. For (d), *n* = 4–5 independent working electrodes; for (e), *n* = 3 independent working electrodes.

Building on the dual‐electrode concepts, we establish a general small‐molecule sensing platform that uses individually functionalized interdigitated electrode (IDE) arrays to overcome the kinetic constraints inherent to earlier designs. Through selective SAM removal and rational spatial engineering, two working electrodes were independently modified with distinct sensing elements: one for target‐induced signal DNA release and the other for capture and readout. This configuration reduces background current and facilitates mass transport, enabling robust transduction of small‐molecule binding events into dual‐channel electrochemical outputs (Figure 1). The platform allows rapid (≤ 30 min), selective detection of model small molecules, demonstrated here with dopamine and cortisol, in aCSF, serum, and saliva, using only 30 µL of sample. We demonstrate physiologically relevant detection in minimally processed biofluids, highlighting the potential of this system for practical POC applications.

## 2 Results and Discussion

### 2.1 Working Principle and Validation of the Dual‐Channel Aptamer Sensing Platform

To realize complementary signal readouts upon target recognition, we engineered a dual‐channel aptamer sensing platform based on an IDE chip (Figure 1a,c), where each working electrode is tailored for signal probe release or capture. Specifically, the two working electrodes (WE1 and WE2) operate in opposing signal transduction modes: WE1 generates a signal‐off response *via* target‐induced strand displacement, while WE2 produces a signal‐on response through recapture of the released redox‐labeled DNA probe (Figure 1b). This complementary dual‐channel configuration minimizes false interpretation due to baseline drift or background interference through internal referencing. In addition to the dual‐channel logic, the platform introduces two important key designs: (1) an IDE architecture that defines a short transport distance (e.g., 100 µm) enabling efficient probe transfer between WE1 and WE2, and (2) selective surface reactivation, which allows precise spatial functionalization of each electrode with distinct sensing chemistries.

As an initial proof‐of‐concept, we used dopamine as a model small‐molecule target due to its essential roles in neurotransmission, clinical relevance to neurological disorders [1, 29], and the availability of a well‐characterized aptamer with high affinity and specificity (*K_d_* =  150 nM) [30]. Time‐resolved square wave voltammetry (SWV) measurements in aCSF confirmed the intended dual‐channel operation: upon exposure to 100 nM dopamine, WE1 exhibited a time‐dependent decrease in peak current over 120 min (Figure 1d), consistent with the progressive release of the MB‐cDNA from the electrode surface *via* target‐induced strand displacement. Conversely, WE2 exhibited a corresponding signal increase (Figure 1e), attributed to recapture of the released probe by a complementary capture probe (CAP) immobilized on WE2. The signal at WE2 approached saturation after approximately 60 min. Control experiments in dopamine‐free aCSF showed negligible signal change at both electrodes, confirming target‐specific responses and minimal nonspecific interactions or crosstalk between WE1 and WE2. These results validate our interdigitated dual‐channel design for bidirectional signal transduction in biological media.

### 2.2 Sequence Optimization *via* Fluorescence Assay

**FIGURE 2:**
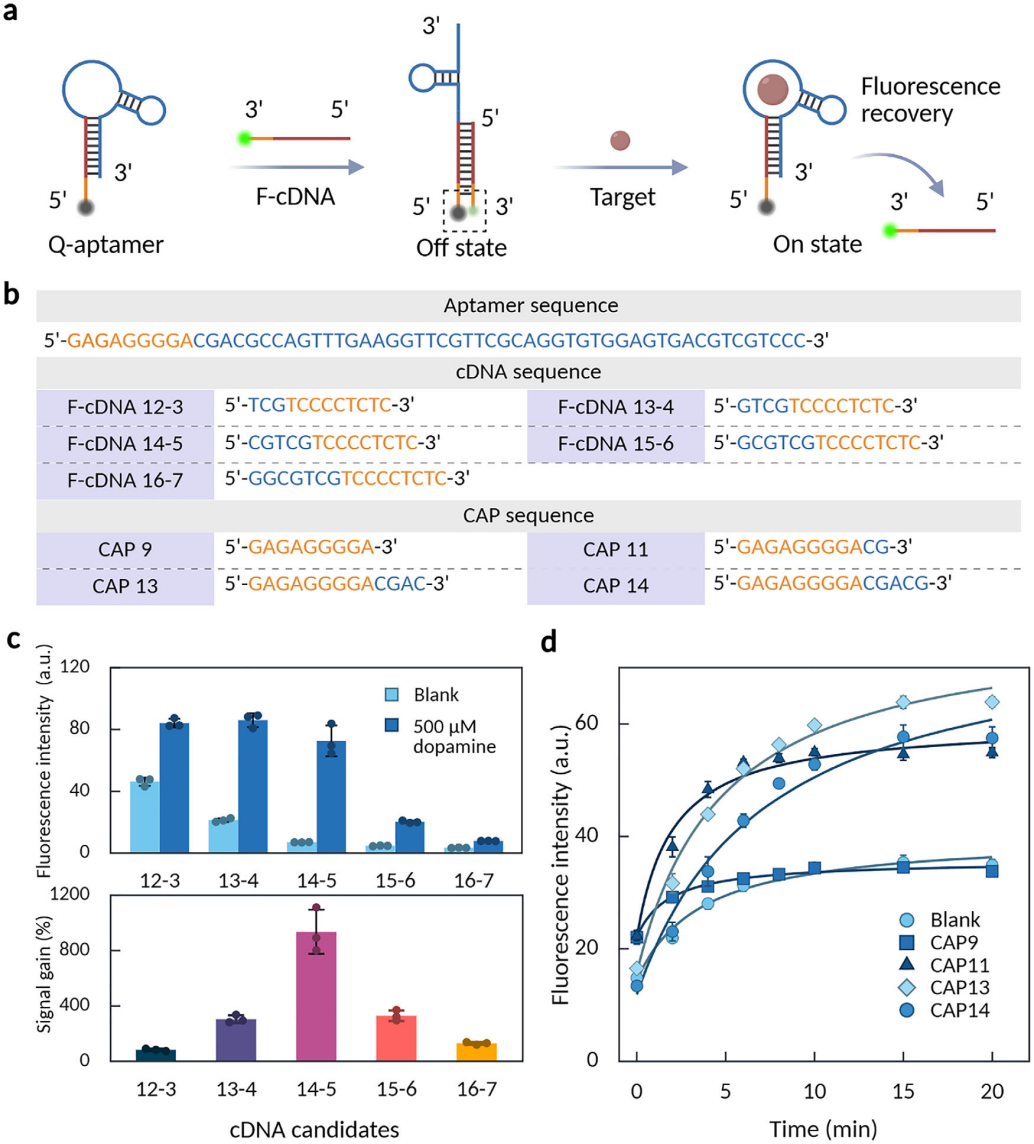
Fluorescence assay for the optimization of cDNA and CAP sequences. (a) Schematic illustration: the quencher‐labeled aptamer (Q‐aptamer) hybridizes with fluorophore‐labeled cDNA (F‐cDNA) to form a quenched complex (off‐state). Upon target binding, the aptamer undergoes a conformational change that displaces the F‐cDNA, restoring fluorescence (on‐state). (b) Summary table of sequences used in the optimization experiments, including the Q‐aptamer, F‐cDNA variants, and CAP. In the aptamer sequence, the blue region denotes the original aptamer domain, while the orange region indicates the extended sequence. Identically colored regions are complementary. (c) Screening of F‐cDNA candidates based on fluorescence intensity in the presence and absence of the target (top), and corresponding signal gain (bottom) to identify optimal displacement dynamics. Fluorescence values were recorded at 520 nm after 2 min of incubation with 500 µM dopamine as the model analyte. (d) Kinetics of F‐cDNA displacement from the duplex by different CAP sequences over a 20‐min period, with fluorescence intensity measured at 0, 2, 4, 6, 8, 10, 15, and 20 min. Data in (c) and (d) are presented as mean ± SD from three independent measurements (*n* = 3).

To enable efficient strand displacement and selective capture for dual‐channel signal transduction, we systematically optimized the sequences of the complementary DNA (cDNA) and the CAP using a fluorescence‐based assay. In this assay, a quencher‐labeled aptamer (Q‐aptamer) was pre‐hybridized with a fluorophore‐labeled cDNA (F‐cDNA), forming a quenched duplex. Upon target binding, the aptamer undergoes a structural rearrangement that releases the F‐cDNA, restoring fluorescence (Figure 2a, Figure S1a). To identify a strand with optimal displacement performance, six F‐cDNA variants (14–1 to 14–6) with different hybridization strengths were designed (Figure 2b). Among them, F‐cDNA variant 14–5 yielded the highest fluorescence signal gain upon dopamine addition (940%, Figure 2c, Table S1), indicating an optimal balance between duplex stability and efficient target‐triggered displacement. The concentration of F‐cDNA was subsequently optimized to ensure efficient duplex formation with the Q‐aptamer (Figure S1b). The selected Q‐aptamer/F‐cDNA 14–5 duplex exhibited a clear, concentration‐dependent fluorescence response to dopamine in a range of 0–500 µM (Figure S1c,d). Notably, fluorescence intensity reached its maximum within 2 min after target addition, confirming the rapid and efficient displacement behavior of this sequence (Figure S1e). The F‐cDNA variant 14–5 was thus selected for subsequent use.

To ensure selective post‐displacement capture of cDNA without destabilizing the aptamer/cDNA duplex prior to target binding, we evaluated several candidate CAP sequences by introducing them into pre‐formed duplex complexes. If a CAP strand exhibited stronger hybridization affinity to F‐cDNA than the aptamer itself, it could prematurely displace the F‐cDNA from the duplex, leading to unwanted signal recovery and compromised sensing performance. As shown in Figure 2d, incubation with CAP11, CAP13, or CAP14 for 20 mins led to a marked fluorescence increase, indicating disruption of the original Q‐aptamer/F‐cDNA complex. Such nonspecific displacement could interfere with subsequent surface immobilization processes. In contrast, CAP9 yielded a fluorescence signal comparable to the blank, confirming that it did not disturb the duplex under these conditions. To verify its capture ability, a quencher‐labeled CAP9 was incubated with F‐cDNA, resulting in a concentration‐dependent fluorescence decrease (Figure S1f). These results demonstrate that CAP9 effectively hybridizes with displaced F‐cDNA and functions as a reliable capture probe and was therefore selected for further use.

### 2.3 Surface Engineering and Characterization of the IDE Aptamer Sensing Platform

**FIGURE 3:**
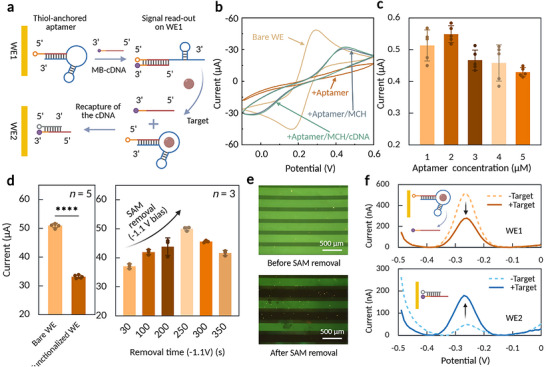
Functionalization and selective self‐assembled monolayer (SAM) removal on the IDE chip for dual‐channel sensing. (a) Schematic illustration of the sensing strategy: On WE1, thiol‐anchored aptamers hybridized with MB‐cDNA are immobilized. Upon target binding, cDNA is released and subsequently recaptured by the capture probe on WE2, enabling a dual‐signal output. (b) CV characterization of the working electrode after sequential functionalization steps. (c) Optimization of aptamer concentration (1–5 µM) based on the SWV peak current following hybridization with 1 µM MB‐cDNA. Based on the maximal current response, 2 µM was selected for subsequent experiments. (d) Left: Comparison of CV peak currents between bare and functionalized electrodes, showing a decreased current due to SAM formation. Right: Optimization of electrochemical SAM removal on WE2 at −1.1 V for varying durations. Peak current increases with extended removal time, saturating at ∼250 s. (e) Fluorescence images before and after SAM removal using SYBR Gold staining, a cyanine dye that exhibits strong fluorescence enhancement upon binding to DNA, demonstrating selective removal of the DNA‐functionalized SAM from WE2. (f) SWV responses of WE1 (top) and WE2 (bottom) in the absence and presence of 100 nM dopamine, exhibiting a signal‐off response at WE1 and a signal‐on behavior at WE2 due to target‐induced cDNA displacement and recapture (see inset schematics). Data in (c) and (d) are presented as mean ± SD, with *n* = 5 for (c) and *n* = 5 (left) and *n* = 3 (right) for (d). Statistical analysis was performed using an unpaired two‐sample *t*‐test; significant differences in CV peak currents between bare and functionalized WEs were observed (***p < 0.0001). CV measurements in (b) and (d) were conducted in PBS supplemented with 100 mM KCl and 5 mM [Fe(CN)_6_]^4^^−^/[Fe(CN)_6_]^3^^−^ (1:1).

To establish the dual‐channel sensing platform (Figure 3a), IDE chips were fabricated by sputtering Cr/Au onto glass substrates, followed by rapid prototyping *via* UV‐laser ablation (Figure S2a, Figure S3a). The final chip layout is shown in Figure S3b,c, with each IDE comprising WE1 and WE2. After electrochemical cleaning, cyclic voltammetry (CV) showed consistent peak currents from 40 WEs, demonstrating the scalability and reproducibility of the fabrication process (Figure S4a).

Both WEs were initially functionalized identically (Figure S2b). Characterization *via* CV confirmed successful stepwise surface modification (Figure 3b). Immobilization of the thiolated aptamer hindered interfacial electron transfer, as evidenced by a marked decrease in CV peak current. Subsequent backfilling with 6‐mercapto‐1‐hexanol (MCH) reduced nonspecific DNA surface adsorption and facilitated the formation of a well‐ordered monolayer, leading to a partial recovery of the redox signal [31]. Hybridization with MB‐cDNA did not induce significant changes in CV, but successful modification was confirmed by the appearance of a characteristic MB redox peak in SWV (Figure S5). Consistent functionalization curves across eight working electrodes from four independent chips demonstrated the reproducibility of the surface modification protocol (Figure S6). The effect of the aptamer concentration was then investigated, with 2 µM aptamer yielding the strongest SWV signal upon hybridization with 1 µM MB‐cDNA (Figure 3c). This concentration corresponds to a surface density of (4.01 ± 1.51) × 10^11^ molecules/cm^2^, consistent with a low packing density that is known to promote hybridization efficiency [32]. Other parameters, including MCH concentration, hybridization time, and MB‐cDNA concentration, were also investigated to obtain optimal sensing performance (Figure S7).

The interdigitated layout inherently exposes both electrodes to the same chemical modifications during fabrication, leading to identical SAM formation. To decouple the sensing elements, we employed electrochemical desorption at −1.1 V to selectively remove the SAM from WE2, thus enabling subsequent probe immobilization on only one electrode [33, 34]. The CVs showed that the redox current significantly decreased upon full modification compared to the bare WE, confirming the formation of a SAM (Figure 3d **, left**). To reactivate WE2, reductive desorption was applied at −1.1 V for varying durations. The redox current gradually increased with desorption time, plateauing at 250 s, beyond which no significant recovery was observed (Figure 3d, **right**, Figure S8a), indicating complete and stable SAM removal. This result was further supported by the disappearance of the MB redox signal in SWV (Figure S8b). The interdigitated electrode spacing of 100 µm ensured that localized desorption did not affect neighboring electrodes, thereby preserving the SAM of WE1 (Figure S8c) [35]. Successful SAM assembly and subsequent electrochemical removal were confirmed by atomic force microscopy (AFM) (Figure S9) and corroborated by SYBR Gold staining, a fluorescent dye that exhibits > 1000‐fold signal enhancement upon binding to nucleic acids (Figure 3e) [36]. Fluorescence imaging revealed a loss of signal on WE2 following SAM removal, while the neighboring WE1 retained high fluorescence, confirming that the desorption was spatially confined and did not affect neighboring electrodes. Following full functionalization, incubation with 100 nM dopamine induced the expected complementary signal behavior: a decrease in the SWV peak current at WE1 due to MB‐cDNA release, and the emergence of a clear MB redox peak at approximately –0.28 V at WE2 due to strand recapture (Figure 3f). These results validate the signal conversion mechanism central to our sensing platform.

### 2.4 Selective Dopamine Detection in aCSF

**FIGURE 4:**
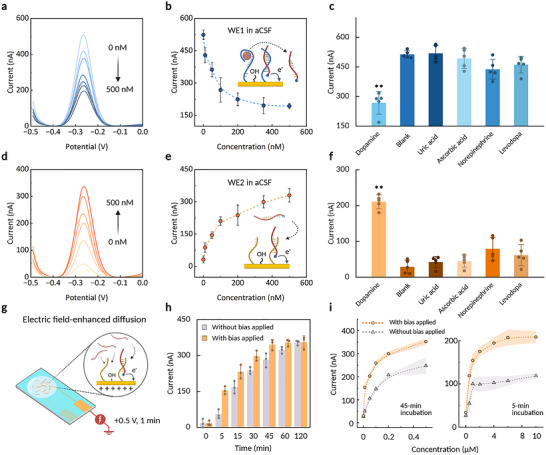
Detection of dopamine in aCSF using the IDE chip with an electric field‐assisted strategy for rapid sensing. (a) SWV curves from WE1 in aCSF upon exposure to increasing concentrations of dopamine (0–500 nM). (b) Calibration curve based on SWV peak current at WE1, showing a concentration‐dependent signal decrease. (c) Selectivity test of WE1 against electroactive and structurally similar interferents (uric acid, ascorbic acid, norepinephrine, levodopa) and blank buffer. (d) SWV curves of WE2 in aCSF with dopamine concentrations from 0 to 500 nM. (e) Corresponding calibration curve at WE2, exhibiting a signal‐on response with increasing dopamine concentrations. (f) Selectivity test of WE2 under the same conditions as (c), showing specificity for dopamine versus nonspecific molecules. (g) Schematic illustration of the electric field‐assisted diffusion strategy: a +0.5 V bias was applied for 1 min to enhance MB‐cDNA migration toward WE2 prior to incubation. (h) Time‐dependent signal enhancement at WE2 with 500 nM dopamine, with and without electric field application. (i) Concentration–response curves for dopamine detection following 45 min (left) and 5 min (right) incubation, demonstrating improved sensitivity and accelerated detection under biased conditions. All target incubations in (a–f) were performed for 2 h at room temperature. In (c) and (f), dopamine and all other analytes were tested at 100 nM. Data are presented as mean ± SD. For (b), (c), (e), and (f), *n* = 5 independent measurements; for (h) and (i), *n* = 3. Statistical analysis was performed using an unpaired two‐sample *t*‐test; significant differences between dopamine and other nonspecific molecules were observed (**p < 0.01).

To evaluate the performance of our dual‐channel sensing platform in a physiologically relevant environment, we tested its ability to detect dopamine in aCSF. At WE1, SWV measurements revealed a concentration‐dependent decrease in peak current upon dopamine addition (0–500 nM), indicating the target‐induced release of MB‐cDNA from the aptamer duplex (Figure 4a). The corresponding calibration curve demonstrated a clear monotonic decay with increasing dopamine concentrations (Figure 4b). Notably, the signal plateaued at 500 nM, suggesting saturation of releasable MB‐cDNA at WE1. In contrast, WE2 exhibited a signal‐on response: the released MB‐cDNA was recaptured by the complementary CAP, leading to an increased SWV current with rising dopamine concentration (Figure 4d). This trend was validated by the corresponding calibration curve (Figure 4e). Linear regression of the calibration curves in their dynamic ranges yielded limits of detection (LOD) of 18 nM for WE1 and 3 nM for WE2, showing that the signal‐on channel enabled a lower LOD. To assess selectivity, we tested structurally similar neurotransmitters and electroactive interferents commonly found in biological matrices, including uric acid, ascorbic acid, norepinephrine, and levodopa. Among these, only dopamine produced a significant signal change at WE1, confirming selective target recognition (Figure 4c, Figure S10a). The same interferents caused negligible signal responses at WE2, further verifying the specificity of the dual‐channel system for dopamine versus nonspecific molecules (Figure 4f, Figure S10b).

Although the 120‐min incubation time used in the above experiments ensured robust signal generation, such a duration remains suboptimal for POC applications where rapid turnaround is important. To address this limitation, we explored a bias‐assisted diffusion approach to increase the transport of released MB‐cDNA toward the capture electrode (WE2). A +0.5 V bias was applied to WE2 for 1 min prior to incubation (Figure 4g). For steady state in the absence of Faradaic reactions, the electric field is confined to the electrical double layer. Yet, residual background and charging currents at the interdigitated electrode allow partial field penetration beyond the Debye length. This effect can drive electrophoretic transport and/or induce electroosmotic flow, thereby assisting the diffusive transport of the negatively charged MB‐cDNA strands released from the electrode surface. Time‐resolved SWV measurements confirmed that applying a +0.5 V bias led to an enhancement in current response at WE2 over a 120‐min period. Compared to passive diffusion, bias‐assisted detection produced a faster increase in signal, particularly within the first 30–60 min. However, after 2 h, the signals obtained from both conditions converged, indicating that the field primarily accelerates early‐stage probe transport (Figure 4h).

Building on these findings, we further evaluated the impact of this approach under shortened incubation durations. Two time points—45 and 5 min—were selected for evaluation. A 45‐min incubation represents an ideal time frame for POC assays without compromising sensitivity, while a 5‐min duration was chosen to probe the upper limit of assay speed, as initial tests already showed notable signal differences under this condition. At 45 min, the calibration curve obtained with bias assistance showed increased current responses and improved sensitivity across the entire tested range (0–500 nM), compared to the no‐bias condition (Figure 4i **, left**). Notably, even under 5‐min incubation, the system still showed a concentration‐dependent signal in a higher concentration range (0–10 µM) when field enhancement was applied, whereas the response without bias remained weak and poorly resolved (Figure 4i **, right**). While this shorter incubation time compromised resolution, the results demonstrate the feasibility of rapid detection using our platform. Depending on the expected target concentration range, the incubation time can be adjusted accordingly to balance assay speed and sensitivity.

Based on the bias‐assisted acceleration of signal generation and the feasibility of shortened incubation times, we next investigated how the inter‐electrode distance of the IDE chip influences MB‐cDNA transport between the two working electrodes. For an IDE chip with 100 µm spacing and a reported diffusion coefficient of a 14‐nucleotide ssDNA oligomer in TE buffer at 20°C (*D* = 1.48 × 10^−10^ m^2^ s^−1^) [37], the diffusion time between WE1 and WE2 can be estimated as ∼0.6 min (*t*  = *x* ^2^/2*D* ). This result is an estimate derived from a simplified 1‐D diffusion model considering the shortest distance between the two electrodes. In the actual case, probe transport also occurs along significantly larger trajectories in 3‐D and can be influenced by multiple factors. As a result, the sensor response can be much slower than predicted by the idealized 1‐D diffusion, consistent with the experimentally observed kinetics.

Experiments performed with IDE chips featuring different inter‐electrode gaps showed that increasing the gap leads to longer transport times, regardless of whether a bias is applied. As shown in Figure S11, under bias‐assisted conditions in the experiment results, signal improvement is most pronounced during the early stages of incubation (within the first 60 min) for gap distances of 100 and 200 µm. In contrast, for larger gaps (500 µm), the bias‐assisted strategy provided negligible improvement.

To further examine the contributions of diffusive and electrophoretic transport, COMSOL simulations were performed modelling DNA migration induced by a background current of 41 nA, corresponding to the +0.5 V bias applied in the experiments. As shown in Figure S12, while reducing the spacing markedly accelerates probe accumulation at WE2, the applied current produces only a minor electrophoretic effect on MB‐cDNA transport under the simulated conditions. These results suggest that steady‐state electrophoretic migration alone is insufficient to account for the experimentally observed bias‐assisted enhancement, indicating that additional mechanisms (such as electroosmosis) likely contribute to the accelerated signal transduction [38]. Accordingly, based on experimental observations, a 100 µm IDE gap appears to be optimal, providing efficient bias‐assisted transport while maintaining reliable electrode functionalization. Overall, these results demonstrate that closely spaced IDEs enable enhanced probe transport and effective bias‐assisted operation.

We further considered the practical translation of this platform toward POC implementation. In a realistic POC scenario, all surface functionalization steps, including SAM formation, selective electrochemical desorption, and subsequent re‐functionalization, would be integrated into factory‐level chip fabrication. Users would only need to introduce the sample and initiate the measurement, while the bias‐assisted diffusion and electrochemical readout could be executed *via* simple, pre‐programmed protocols integrated into the device interface. To support the feasibility of this workflow, we evaluated the stability of the fully functionalized IDE chips. Sensors stored in PBS at 4°C retained more than 80% of their original target response at both WE1 and WE2 after 7 days (Figure S13). For applications requiring prolonged storage at higher temperatures (e.g., room temperature [≈25°C] or physiological temperature [37°C]), additional stabilization strategies, such as polymer coatings or protective surface modifications, could be explored to improve long‐term robustness [39, 40].

### 2.5 Rapid Detection of Cortisol in Human Serum and Saliva

To demonstrate the generalizability of our sensing platform, we extended its application to the detection of cortisol—a clinically relevant small‐molecule biomarker associated with stress‐related physiological responses. The POC monitoring of cortisol holds high potential for personalized stress assessment and endocrine health management [41, 42]. Using a previously reported cortisol‐specific aptamer (*K_d_* =  100 nM) [43], we optimized the cDNA strand and implemented the same signal conversion mechanism as for dopamine (Figure S14a,b, Table S2).

**FIGURE 5:**
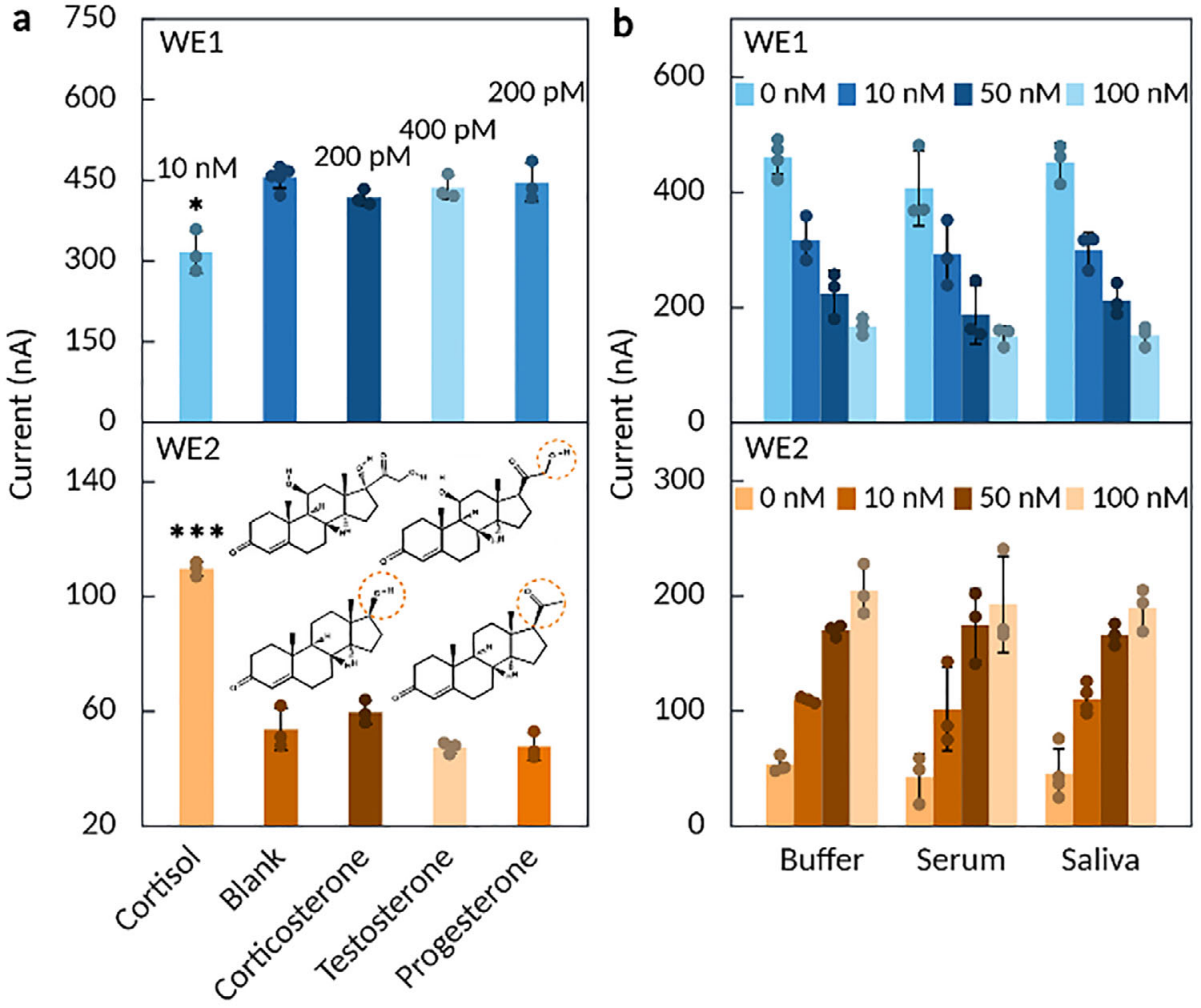
Cortisol specificity and validation of the dual‐channel sensing platform in different biofluids. (a) Selectivity evaluation of WE1 and WE2 against structurally related steroids (corticosterone, testosterone, and progesterone) and blank buffer. Negligible responses to nontarget confirm the specificity of the platform. Molecule concentrations were chosen based on their relevant physiological levels. (b) Detection of cortisol using the dual‐channel platform, showing current responses at WE1 and WE2 in buffer, serum, and saliva with different increasing cortisol concentrations (0, 10, 50, and 100 nM). Data are presented as mean ± SD (*n =* 3–5). All measurements were performed using electric field‐assisted diffusion and a 30‐min incubation. Statistical analysis was performed using an unpaired two‐sample *t*‐test; significant differences between cortisol and other groups were observed (*p < 0.05, ***p < 0.001).

We next implemented the optimized sequence and evaluated the cortisol response in buffer (PBS containing Mg^2+^ and Ca^2+^), followed by selectivity testing against structurally related interferents (progesterone, corticosterone, and testosterone) using the proposed platform at physiologically relevant concentrations (Figure 5a) [42]. Only cortisol induced statistically significant signal changes.

We then investigated whether the current response could be maintained in different biofluids, including serum and saliva. For serum measurements, samples were diluted 1:1 with PBS. In the case of saliva, cortisol levels follow a circadian rhythm and are relatively high in the morning. To minimize endogenous background, saliva samples were therefore collected at 9 p.m., when cortisol levels approach their physiological minimum, and diluted to 30% prior to analysis. Reported evening salivary cortisol concentrations of ∼1–3 nM [44, 45] were thereby reduced to the picomolar range, below the cortisol sensor LOD (∼3 nM for cortisol at WE2).

Under these conditions, the platform exhibited comparable concentration‐dependent signal changes at both WE1 and WE2 in buffer, serum, and saliva, with slightly increased noise in serum likely arising from variations in ionic strength, viscosity, and nonspecific interactions (Figure 5b). Importantly, the platform retained similar sensing behavior across different biofluids, showing the generalizability and robustness of the dual‐electrode signal conversion strategy. We note that this performance is also inherently aptamer‐dependent, as successful operation across biofluids relies on the aptamer maintaining binding affinity and conformational switching under fluid‐specific ionic conditions; not all aptamers exhibit comparable tolerances [46, 47]. While direct detection in undiluted samples remains challenging due to basal levels of the analyte, diluted samples preserve a low sample‐processing burden and remain compatible with POC translation. Our dilution factor is comparable to that of the commercial COVID‐19 rapid test [48]. Taken together, these results demonstrate that the dual‐channel aptamer sensor enables rapid (30 min) and robust detection of small molecules in multiple biofluids, highlighting the versatility of the platform.

## 3 Conclusion

In summary, we present a dual‐channel electrochemical aptamer‐based sensing platform that achieves signal transduction by spatially separating recognition and readout events on an IDE chip. Different surface modifications were applied to the two IDE electrodes: one serving as the release site for MB‐cDNA upon target‐induced strand displacement (WE1), and the other functioning as a capture site for recapturing the released probes (WE2). This configuration enables differential signal outputs at both electrodes while substantially reducing the influence of background current compared to conventional single‐electrode designs. As a proof‐of‐concept, dopamine was first selected as the model analyte. A fluorescence‐based assay was employed to identify the optimal cDNA sequence for signal generation. The optimized sensing construct enabled selective detection of dopamine in aCSF across physiologically relevant concentrations, with a LOD of 18 nM at WE1 and 3 nM at WE2. The platform was then extended to cortisol detection, demonstrating concentration‐dependent responses in minimally processed serum and saliva samples without requiring extensive preprocessing or reconfiguration.

Although the IDE platform enables reliable detection of dopamine and cortisol in diluted biofluid samples, its direct application to undiluted complex media such as plasma or whole blood remains challenging. These limitations originate from nonspecific adsorption of biomacromolecules and degradation or displacement of the SAM under highly fouling conditions [15, 49]. Future work will therefore focus on systematically evaluating the platform's long‐term operational stability in complex biofluids, potentially incorporating antifouling strategies to reduce nonspecific binding and signal drift [20, 50]. Recent studies have demonstrated promising approaches, including polymer‐based coatings that use different surface‐charge‐mediated backfilling strategies [51, 52, 53]. In this context, the present platform, with independently functionalized WE1 and WE2 chemistries, could further expand this approach by integrating surface‐charge–mediated selectivity engineering. For example, implementing SAMs with distinct charge characteristics on the release and capture channels could further optimize probe release and recapture efficiency, thereby enhancing sensitivity.

Beyond biochemical stability, electrostatic screening will also become a key limitation, especially for sensing in high‐ionic‐strength biofluids. In translational scenarios, continuous monitoring of small‐molecule biomarkers in alternative biofluids such as sweat or interstitial fluid can further exacerbate the impact of electrostatic limitations [12]. Recent work has highlighted that nanostructured electrode architectures can partially mitigate Debye‐length limitations by positioning the redox event within the electrochemical double layer [54]. In this context, the IDE configuration offers additional design flexibility, including further reduction of interdigitated finger spacing and the introduction of controlled electrode roughness or hierarchical nanostructures to enhance signal transduction under physiologically relevant conditions.

Finally, while this work demonstrates that the sensing strategy can be adapted to different small‐molecule analytes by substituting the aptamer sequence, performance remains aptamer‐dependent, as not all aptamers retain matrix tolerance outside the environments in which they were selected. In addition, variability in aptamer *K_d_* values necessitates optimization of sensing parameters to achieve physiologically relevant linear detection ranges and sensitivity. These considerations become even more critical as the platform extends toward continuous, reversible monitoring, which introduces additional requirements on sensor kinetics. In this regard, recent modular intramolecular strand‐displacement designs have demonstrated reversible, real‐time sensing capabilities using optical readouts [55, 56]. Although developed for an alternative transduction platform, such designs provide inspiration for electrochemical sensing methods. Integrating analogous reversible probe designs with spatially separated release and capture channels may offer a general strategy for enabling dynamic probe exchange and time‐resolved signal transduction, supporting the future development of translational POC biosensing platforms.

## Author Contributions

**Senyao Wang**: conceptualization, investigation, writing – original draft, methodology, visualization, formal analysis, funding acquisition. **Ali Elmorsy**: methodology, validation, software, writing – original draft, data curation. **Defne Tüzün**: investigation, data curation, writing – review and editing, resources. **Lilly Schmidt**: data curation, investigation. **Sebastian Freko**: conceptualization, writing – review and editing. **Lukas Hiendlmeier**: resources. **Chen Wang**: investigation, writing – review and editing, resources. **Alonso I. Romero**: resources. **George A. Boustani**: resources. **Hu Peng**: conceptualization, resources. **Berna Özkale**: writing – review and editing, supervision, resources. **Nako Nakatsuka**: writing – review and editing, funding acquisition, supervision, resources, conceptualization. **Bernhard Wolfrum**: funding acquisition, conceptualization, writing – review and editing, supervision, resources, project administration.

## Conflicts of Interest

The authors declare no conflicts of interest.

## Supporting information




**Supporting File**: anie72577‐sup‐0001‐SuppMat.docx.

## Data Availability

The data that support the findings of this study are available from the corresponding author upon reasonable request.

## References

[1] R. A. Wise , “Dopamine, Learning and Motivation,” Nature Reviews Neuroscience 5 (2004): 483–494, 10.1038/nrn1406. 15152198

[2] E. R. de Kloet , M. Joëls , and F. Holsboer , “Stress and the Brain: From Adaptation to Disease,” Nature Reviews Neuroscience 6 (2005): 463–475, 10.1038/nrn1683. 15891777

[3] Y. Dai and C. C. Liu , “Recent Advances on Electrochemical Biosensing Strategies Toward Universal Point‐of‐Care Systems,” Angewandte Chemie International Edition 58 (2019): 12355–12368, 10.1002/anie.201901879. 30990933

[4] X. Wang , L. Cohen , J. Wang , and D. R. Walt , “Competitive Immunoassays for the Detection of Small Molecules Using Single Molecule Arrays,” Journal of the American Chemical Society 140 (2018): 18132–18139, 10.1021/jacs.8b11185. 30495929

[5] A. Frutiger , A. Tanno , S. Hwu , R. F. Tiefenauer , J. Vörös , and N. Nakatsuka , “Nonspecific Binding—Fundamental Concepts and Consequences for Biosensing Applications,” Chemical Reviews 121 (2021): 8095–8160, 10.1021/acs.chemrev.1c00044. 34105942

[6] P. Nandhakumar , C. Muñoz San Martín , B. Arévalo , et al., “Redox Cycling Amplified Electrochemical Lateral‐Flow Immunoassay: Toward Decentralized Sensitive Insulin Detection,” ACS Sensors 8 (2023): 3892–3901, 10.1021/acssensors.3c01445. 37734056

[7] S. Kim and M.‐G. Kim , “Automatically Signal‐Enhanced Lateral Flow Immunoassay for Ultrasensitive Salivary Cortisol Detection,” Analytical Chemistry 97 (2025): 2707–2713, 10.1021/acs.analchem.4c04700. 39895203 PMC11822745

[8] J. L. Powers , K. D. Rippe , K. Imarhia , A. Swift , M. Scholten , and N. Islam , “A Direct, Competitive Enzyme‐Linked Immunosorbent Assay (ELISA) as a Quantitative Technique for Small Molecules,” Journal of Chemical Education 89 (2012): 1587–1590, 10.1021/ed2005505.

[9] A. Adumitrăchioaie , M. Tertiș , M. Suciu , F. Graur , and C. Cristea , “A Novel Immunosensing Platform for Serotonin Detection in Complex Real Samples Based on Graphene Oxide and Chitosan,” Electrochimica Acta 311 (2019): 50–61, 10.1016/j.electacta.2019.04.128.

[10] Y. Liu , L. Zhan , Z. Qin , J. Sackrison , and J. C. Bischof , “Ultrasensitive and Highly Specific Lateral Flow Assays for Point‐of‐Care Diagnosis,” ACS Nano 15 (2021): 3593–3611, 10.1021/acsnano.0c10035. 33607867

[11] P. Peng , C. Liu , Z. Li , et al., “Emerging ELISA Derived Technologies for In Vitro Diagnostics,” Trends in Analytical Chemistry 152 (2022): 116605, 10.1016/j.trac.2022.116605.

[12] C. Ye , H. Lukas , M. Wang , Y. Lee , and W. Gao , “Nucleic Acid‐Based Wearable and Implantable Electrochemical Sensors,” Chemical Society Reviews 53 (2024): 7960–7982, 10.1039/D4CS00001C. 38985007 PMC11308452

[13] J. Wu , H. Liu , W. Chen , B. Ma , and H. Ju , “Device Integration of Electrochemical Biosensors,” Nature Reviews Bioengineering 1 (2023): 346–360, 10.1038/s44222-023-00032-w. PMC9951169 37168735

[14] B. D. Wilson , A. A. Hariri , I. A. P. Thompson , M. Eisenstein , and H. T. Soh , “Independent Control of the Thermodynamic and Kinetic Properties of Aptamer Switches,” Nature Communications 10 (2019): 5079, 10.1038/s41467-019-13137-x. PMC6838323 31699984

[15] K. K. Leung , A. M. Downs , G. Ortega , M. Kurnik , and K. W. Plaxco , “Elucidating the Mechanisms Underlying the Signal Drift of Electrochemical Aptamer‐Based Sensors in Whole Blood,” ACS Sensors 6 (2021): 3340–3347, 10.1021/acssensors.1c01183. 34491055 PMC12038169

[16] A. M. Downs and K. W. Plaxco , “Real‐Time, *In Vivo* Molecular Monitoring Using Electrochemical Aptamer Based Sensors: Opportunities and Challenges,” ACS Sensors 7 (2022): 2823–2832, 10.1021/acssensors.2c01428. 36205360 PMC9840907

[17] S. Li , J. Dai , M. Zhu , et al., “Implantable Hydrogel‐Protective DNA Aptamer‐Based Sensor Supports Accurate, Continuous Electrochemical Analysis of Drugs at Multiple Sites in Living Rats,” ACS Nano 17 (2023): 18525–18538, 10.1021/acsnano.3c06520. 37703911

[18] H. Li , P. Dauphin‐Ducharme , N. Arroyo‐Currás , et al., “A Biomimetic Phosphatidylcholine‐Terminated Monolayer Greatly Improves the In Vivo Performance of Electrochemical Aptamer‐Based Sensors,” Angewandte Chemie International Edition 56 (2017): 7492–7495, 10.1002/anie.201700748. 28371090 PMC5660315

[19] D. Chan , J.‐C. Chien , E. Axpe , et al., “Combinatorial Polyacrylamide Hydrogels for Preventing Biofouling on Implantable Biosensors,” Advanced Materials 34 (2022): 2109764, 10.1002/adma.202109764. PMC9793805 35390209

[20] Y. Chen , K. X. Fu , R. Cotton , et al., “A Biochemical Sensor With Continuous Extended Stability In Vivo,” Nature Biomedical Engineering 9 (2025): 1517–1530, 10.1038/s41551-025-01389-6. PMC12997450 40410556

[21] K. Fu , J.‐W. Seo , V. Kesler , et al., “Accelerated Electron Transfer in Nanostructured Electrodes Improves the Sensitivity of Electrochemical Biosensors,” Advancement of Science 8 (2021): 2102495, 10.1002/advs.202102495. PMC8655170 34668339

[22] A. Stuber , A. Cavaccini , A. Manole , et al., “Interfacing Aptamer‐Modified Nanopipettes With Neuronal Media and *Ex Vivo* Brain Tissue,” American Chemical Society Measurement Science Au 4 (2024): 92–103, 10.1021/acsmeasuresciau.3c00047. 38404490 PMC10885324

[23] A. Stuber and N. Nakatsuka , “Aptamer Renaissance for Neurochemical Biosensing,” ACS Nano 18 (2024): 2552–2563, 10.1021/acsnano.3c09576. 38236046 PMC10832038

[24] N. Arroyo‐Currás , J. Somerson , P. A. Vieira , K. L. Ploense , T. E. Kippin , and K. W. Plaxco , “Real‐Time Measurement of Small Molecules Directly in Awake, Ambulatory Animals,” Proceedings National Academy of Science USA 114 (2017): 645–650, 10.1073/pnas.1613458114. PMC5278471 28069939

[25] R. H. Batchelor , E. M. Dief , A. J. Bonham , and J. J. Gooding , “A Review of Methylene Blue's Interactions With DNA and Their Relevance for DNA‐Based Sensors,” ACS Sensors 10, no. 6 (2025): 3854–3877, 10.1021/acssensors.5c00336. 40443285

[26] Y. Xiao , A. A. Lubin , A. J. Heeger , and K. W. Plaxco , “Label‐Free Electronic Detection of Thrombin in Blood Serum by Using an Aptamer‐Based Sensor,” Angewandte Chemie International Edition 44 (2005): 5456–5459, 10.1002/anie.200500989. 16044476

[27] R. Pandey , D. Chang , M. Smieja , T. Hoare , Y. Li , and L. Soleymani , “Integrating Programmable DNAzymes With Electrical Readout for Rapid and Culture‐Free Bacterial Detection Using a Handheld Platform,” Nature Chemistry 13 (2021): 895–901, 10.1038/s41557-021-00718-x. 34168325

[28] A. Victorious , Z. Zhang , D. Chang , et al., “A DNA Barcode‐Based Aptasensor Enables Rapid Testing of Porcine Epidemic Diarrhea Viruses in Swine Saliva Using Electrochemical Readout,” Angewandte Chemie International Edition 61 (2022): e202204252, 10.1002/anie.202204252. 35567324

[29] S. E. Hyman and R. C. Malenka , “Addiction and the Brain: The Neurobiology of Compulsion and Its Persistence,” Nature Reviews Neuroscience 2 (2001): 695–703, 10.1038/35094560. 11584307

[30] N. Nakatsuka , K.‐A. Yang , J. M. Abendroth , et al., “Aptamer–Field‐Effect Transistors Overcome Debye Length Limitations for Small‐Molecule Sensing,” Science 362 (2018): 319–324, 10.1126/science.aao6750. 30190311 PMC6663484

[31] X. Xu , A. Makaraviciute , S. Kumar , et al., “Structural Changes of Mercaptohexanol Self‐Assembled Monolayers on Gold and Their Influence on Impedimetric Aptamer Sensors,” Analytical Chemistry 91 (2019): 14697–14704, 10.1021/acs.analchem.9b03946. 31650834

[32] J. Zhang , S. Song , L. Zhang , et al., “Sequence‐Specific Detection of Femtomolar DNA via a Chronocoulometric DNA Sensor (CDS): Effects of Nanoparticle‐Mediated Amplification and Nanoscale Control of DNA Assembly at Electrodes,” Journal of the American Chemical Society 128 (2006): 8575–8580, 10.1021/ja061521a. 16802824

[33] L. Niu and W. Knoll , “Electrochemically Addressable Functionalization and Parallel Readout of a DNA Biosensor Array,” Analytical Chemistry 79 (2007): 2695–2702, 10.1021/ac061678l. 17326609

[34] D. Sen and R. A. Lazenby , “Selective Aptamer Modification of Au Surfaces in a Microelectrode Sensor Array for Simultaneous Detection of Multiple Analytes,” Analytical Chemistry 95 (2023): 6828–6835, 10.1021/acs.analchem.2c05335. 37071798

[35] R. Y. Lai , S.‐H. Lee , H. T. Soh , K. W. Plaxco , and A. J. Heeger , “Differential Labeling of Closely Spaced Biosensor Electrodes via Electrochemical Lithography,” Langmuir 22 (2006): 1932–1936, 10.1021/la052132h. 16460130

[36] T. Schlotter , T. Kloter , J. Hengsteler , et al., “Aptamer‐Functionalized Interface Nanopores Enable Amino Acid‐Specific Peptide Detection,” ACS Nano 18 (2024): 6286–6297, 10.1021/acsnano.3c10679. 38355286 PMC10906075

[37] M. Bolten , M. Niermann , and W. Eimer , “Structural Analysis of G‐DNA in Solution: A Combination of Polarized and Depolarized Dynamic Light Scattering With Hydrodynamic Model Calculations,” Biochemistry 38 (1999): 12416–12423, 10.1021/bi990750p. 10493810

[38] L. J. K. Weiß , E. Music , P. Rinklin , M. Banzet , D. Mayer , and B. Wolfrum , “On‐Chip Electrokinetic Micropumping for Nanoparticle Impact Electrochemistry,” Analytical Chemistry 94 (2022): 11600–11609, 10.1021/acs.analchem.2c02017. 35900877

[39] X. Zhou , J. Slaughter , S. Riki , C. C. Kuo , and A. Furst , “Polymer Coating for the Long‐Term Storage of Immobilized DNA,” ACS Sensors 10 (2025): 5019–5026, 10.1021/acssensors.5c00937. 40583695 PMC12306534

[40] Z. Watkins , A. Karajic , T. Young , R. White , and J. Heikenfeld , “Week‐Long Operation of Electrochemical Aptamer Sensors: New Insights Into Self‐Assembled Monolayer Degradation Mechanisms and Solutions for Stability in Serum at Body Temperature,” ACS Sensors 8 (2023): 1119–1131, 10.1021/acssensors.2c02403. 36884003 PMC10443649

[41] Z. Li , D. Luo , Y. Zhang , X. Niu , and H. Liu , “Smart Health Monitoring: Review of Electrochemical Biosensors for Cortisol Monitoring,” Advanced Healthcare Materials 14 (2025): 2404454, 10.1002/adhm.202404454. 40099568

[42] B. Wang , C. Zhao , Z. Wang , et al., “Wearable Aptamer‐Field‐Effect Transistor Sensing System for Noninvasive Cortisol Monitoring,” Science Advances 8 (2022): eabk0967, 10.1126/sciadv.abk0967. 34985954 PMC8730602

[43] K.‐A. Yang , H. Chun , Y. Zhang , et al., “High‐Affinity Nucleic‐Acid‐Based Receptors for Steroids,” ACS Chemical Biology 12 (2017): 3103–3112, 10.1021/acschembio.7b00634. 29083858 PMC6737899

[44] M. Trilck , J. Flitsch , D. Lüdecke , R. Jung , and S. Petersenn , “Salivary Cortisol Measurement—A Reliable Method for the Diagnosis of Cushing's Syndrome,” Experimental and Clinical Endocrinology & Diabetes 113 (2005): 225–230, 10.1055/s-2005-837667. 15891959

[45] M. Jia , W. M. Chew , Y. Feinstein , P. Skeath , and E. M. Sternberg , “Quantification of Cortisol in Human Eccrine Sweat by Liquid Chromatography—Tandem Mass Spectrometry,” Analyst 141 (2016): 2053–2060, 10.1039/C5AN02387D. 26858998 PMC5376102

[46] N. Nakatsuka , J. M. Abendroth , K.‐A. Yang , and A. M. Andrews , “Divalent Cation Dependence Enhances Dopamine Aptamer Biosensing,” American Chemical Society Applied Materials & Interfaces 13 (2021): 9425–9435, 10.1021/acsami.0c17535. PMC7933093 33410656

[47] A. Douaki , A. Stuber , J. Hengsteler , et al., “Theoretical Analysis of Divalent Cation Effects on Aptamer Recognition of Neurotransmitter Targets,” Chemical Communications 59 (2023): 14713–14716, 10.1039/D3CC04334G. 37997814

[48] G. C. K. Mak , P. K. C. Cheng , S. S. Y. Lau , et al., “Evaluation of Rapid Antigen Test for Detection of SARS‐CoV‐2 Virus,” Journal of Clinical Virology 129 (2020): 104500, 10.1016/j.jcv.2020.104500. 32585619 PMC7278630

[49] J. D. Pham , L. C. Fetter , J. Gerson , T. E. Kippin , K. W. Plaxco , and K. K. Leung , “On the Blood Components Contributing to the Drift of Electrochemical Aptamer‐Based Biosensors,” ACS Sensors 10 (2025): 5160–5165, 10.1021/acssensors.5c01267.40591816

[50] J.‐C. Lee , S. Y. Kim , J. Song , et al., “Micrometer‐Thick and Porous Nanocomposite Coating for Electrochemical Sensors With Exceptional Antifouling and Electroconducting Properties,” Nature Communications 15 (2024): 711, 10.1038/s41467-024-44822-1. PMC10853525 38331881

[51] V. Kesler , K. Fu , Y. Chen , et al., “Tailoring Electrode Surface Charge to Achieve Discrimination and Quantification of Chemically Similar Small Molecules With Electrochemical Aptamers,” Advanced Functional Materials 33 (2023): 2208534, 10.1002/adfm.202208534. 36819738 PMC9937077

[52] S. Li , Y. Wang , Z. Zhang , Y. Wang , H. Li , and F. Xia , “Exploring End‐Group Effect of Alkanethiol Self‐Assembled Monolayers on Electrochemical Aptamer‐Based Sensors in Biological Fluids,” Analytical Chemistry 93 (2021): 5849–5855, 10.1021/acs.analchem.1c00085. 33787229

[53] M. P. McGowan , A. J. Trowbridge , J. Reitemeier , K. M. Jordan , and K. X. Fu , “Surface Charge Effects of Monovalent and Zwitterionic Monolayers to Differentiate Structurally Similar Aminoglycosides With Electrochemical Aptamer Biosensors,” Biosensors and Bioelectronics 276 (2025): 117229, 10.1016/j.bios.2025.117229. 39954523

[54] G. F. Huldin , J. Huang , and K. X. Fu , “Nanoconfined Constructs for Electrochemical Aptamer‐Based In Vivo Biosensing,” Current Opinion in Electrochemistry 51 (2025): 101695, 10.1016/j.coelec.2025.101695.

[55] A. A. Hariri , A. P. Cartwright , C. Dory , et al., “Modular Aptamer Switches for the Continuous Optical Detection of Small‐Molecule Analytes in Complex Media,” Advanced Materials 36 (2024): 2304410, 10.1002/adma.202304410. 37975267

[56] C. H. Park , I. A. P. Thompson , S. S. Newman , et al., “Real‐Time Spatiotemporal Measurement of Extracellular Signaling Molecules Using an Aptamer Switch‐Conjugated Hydrogel Matrix,” Advanced Materials 36 (2024): 2306704, 10.1002/adma.202306704. 37947789

