# Supplementary material for: Dual‐Channel Interdigitated Aptamer‐Based Sensors for Rapid Small‐Molecule Detection in Biofluids
Source: Angew Chem Int Ed Engl. 2026 May 14;65(27):e8361141. doi: 10.1002/anie.8361141 (PMC13327560; doi:10.1002/anie.8361141)
Supplement: Supplementary file 1 — Supporting File: anie72577‐sup‐0001‐SuppMat.docx. [file ANIE-65-e8361141-s001.docx]

Supporting Information

**Dual-Channel Interdigitated Aptamer-Based Sensors for Rapid Small-Molecule Detection in Biofluids**

*Senyao Wang,^1,2^ Ali Elmorsy,^2^ Defne Tüzün,^1^ Lilly Schmidt,^1^ Sebastian Freko,^1,4^ Lukas Hiendlmeier,^1^ Chen Wang,^3^ Alonso Ingar Romero,^1^ George Al Boustani,^1^ Hu Peng,^1^ Berna Özkale,^3^ Nako Nakatsuka,^2*^ Bernhard Wolfrum^1*^*

^1^Neuroelectronics, Munich Institute of Biomedical Engineering, Department of Electrical Engineering, TUM School of Computation, Information and Technology, Technical University of Munich, 85748 Garching, Germany

^2^Laboratory of Chemical Nanotechnology, École Polytechnique Fédérale de Lausanne (EPFL), 1202 Geneva, Switzerland

^3^Microrobotic Bioengineering Lab (MRBL), Department of Electrical Engineering, TUM School of Computation, Information and Technology, Technical University of Munich, 85748 Garching, Germany

^4^Department of Medicine I, Cardiology, Angiology, Pneumology, Klinikum rechts der Isar, TUM School of Medicine and Health, Technical University of Munich, 81675 Munich, Germany

E-mail: bernhard.wolfrum@tum.de; nako.nakatsuka@epfl.ch

**Materials and reagents**

Materials and reagents were obtained from Sigma-Aldrich (St. Louis, MO, USA) unless otherwise noted. SYBR Gold nucleic acid gel stain was purchased from Thermo Fisher Scientific (Invitrogen, Carlsbad, CA, USA). Oligonucleotides with Dabcyl, 6-FAM, Thiol-C6, and methylene blue modifications (sequences listed in **Table S3**) were synthesized and HPLC-purified by Biomers.net (Ulm, Germany). All DNA sequences were resuspended in PBS (containing 2 mM MgCl₂ and 1 mM CaCl₂) and stored at −20 °C until use. Deionized water (conductivity 0.054 μS/cm) was prepared using a Berry Pure purification system (Berrytec, Harthausen, Germany) and used for all buffer and solution preparations. The compositions of PBS (supplemented with 2 mM MgCl_2_ and 1 mM CaCl_2_), artificial cerebrospinal fluid (aCSF), and PBS containing 5 mM K₄[Fe(CN)₆]/K₃[Fe(CN)₆] (1:1) and 0.1 M KCl used in this study are summarized in **Table S4**.

**Fluorescence assay**

Target displacement experiments were carried out using a fluorescence assay to identify the optimal cDNA sequences. A detailed protocol is provided in **Figure S15**. Briefly, 10 μL of each cDNA candidate was mixed with 10 μL of aptamer solution, heated at 95 °C for 5 min, and then cooled to room temperature for 30 min to allow duplex formation. Subsequently, target solutions at different concentrations (20 μL) were added, and the fluorescence intensity was measured using a microplate reader (Thermo Fisher Scientific, USA) in a 384-well plate format with a total volume of 40 μL. For the capture probe (CAP) displacement experiments, the same protocol was followed, except that the target solution was replaced with 20 μL of 1 μM CAP sequences at different variants. All measurements were performed in triplicate. Fluorescence spectra were recorded at an excitation wavelength of 488 nm and an emission range of 510−650 nm. The fluorescence signal gain was calculated as:

$$Signal gain \left( \% \right)=\frac{F_{target}-F_{blank}}{F_{blank}}\times100\%$$

Where $F_{target}$ is the fluorescence intensity after target addition, and $F_{blank}$ is the fluorescence intensity in the absence of target.

**Fabrication of the IDE chip**

The fabrication steps of the IDE chips are illustrated in **Figure S2a**. The IDE chip was fabricated on 3-inch borosilicate glass wafers (500 µm thickness; MicroChemicals, Ulm, Germany). A 10 nm Cr adhesion layer followed by a 100 nm Au layer was deposited by sputtering using a vacuum magnetron sputtering system (nanoPVD, Moorfield Nanotechnology, UK). The Au electrodes were patterned using a nanosecond pulsed laser scanner (scan speed: 500 mm/s, power: 100% (3W), pulse frequency: 40 kHz; MD-U1000, Keyence, Japan). The wafers were then diced into individual chips (13 mm × 17 mm) using a diamond cutter (MTI Corporation, Richmond, CA, USA). The diced chips were cleaned by 5 min sonication in acetone followed by isopropanol. To create a reservoir, a glass ring (inner diameter: 10 mm; outer diameter: 12 mm; height: 10 mm) was bonded onto each chip using polydimethylsiloxane (PDMS), forming a reservoir. The assembled chips were cured at 50 °C for 1 h to solidify the PDMS. Finally, the chips were rinsed with deionized water and dried under N₂ prior to use.

**Selective functionalization of the IDE chip**

The selective functionalization steps are illustrated in **Figure S2b**. Before functionalization, the IDE chips were electrochemically cleaned by cyclic voltammetry (CV) in 0.1 M H_2_SO_4_ (potential range: 0–1.5 V; scan rate: 100 mV/s; 20 scans). Subsequently, both working electrodes (WE1 and WE2) were incubated overnight with thiolated aptamers (2 µM, 30 µL) for covalent immobilization. To minimize nonspecific adsorption, the electrodes were treated with 6-mercapto-1-hexanol (MCH, 5 mM, 30 µL) for 1 h, effectively backfilling unoccupied sites on the gold surface. Following this step, MB-cDNA (1 μM, 30 μL) was hybridized with the immobilized aptamer for 1.5 h to form a duplex structure. Selective desorption of the self-assembled monolayer (SAM) on WE2 was achieved by applying −1.1 V for 250 s, thereby exposing the bare Au surface.^[1, 2]^ The IDE chip surface was then incubated with CAP (2 µM, 30 µL) for 2 h , followed by backfilling with MCH (5 mM, 30 µL) for 1 h to complete the biosensor assembly. All incubations were conducted at room temperature under static, dark conditions. Following each modification step, the chips were thoroughly rinsed with deionized water and dried under N₂.

**Quantification of surface aptamer densities**

The surface density of the immobilized aptamer probes is a critical parameter that influences both the capture efficiency and signal output of the sensor system. In this work, we quantified the aptamer loading density on the WE surface using a chronocoulometric method based on previous studies.^[3, 4]^ We first determined the electrochemically active surface area ($A$) of each WE by integrating the charge associated with the gold oxide (AuOₓ) reduction peak in CV (**Figure S4b**). The CV was conducted in 0.1 M H₂SO₄ with a potential from 0 V to +1.5 V at a scan rate of 100 mV/s. The reduction charge $Q_{AuO_{x}}$was then divided by the surface charge density required for a monolayer formation of AuOₓ (386 µC/cm²), yielding an active surface area ($A$). Subsequently, chronocoulometry (CC) was performed in 10 mM TE buffer containing 100 µM [Ru(NH₃)₆]³⁺ (RuHex), which electrostatically binds to the phosphate backbone of DNA. The CC curves (Q vs. t¹ᐟ²) were recorded using a potential step from 0.1 V to −0.4 V with a pulse width of 0.25 s and a sample interval of 0.002 s. The intercepts at t = 0 without and with RuHex were used to extract the capacitive and total charges ($Q_{dl}$ and $Q_{total}$), and the Faradaic component corresponding to DNA-bound RuHex, representing the surface-confined single-stranded aptamer ($Q_{ss}$), was calculated as:

$$Q_{ss}=Q_{total}-Q_{dl}$$

The DNA surface density was calculated using the following equation:

$$\Gamma=\frac{Q_{ss}N_{A}}{nFA}\cdot\frac{z}{m}$$

where $Q$ is the RuHex-related charge, $N_{A}$​ is Avogadro’s constant, $F$ is Faraday’s constant, $A$ is the active electrode area, $z$ = +3 is the charge number per RuHex molecule, $m$ is the number of nucleotides per strand, and $n$=1 is the number of electrons transferred per RuHex molecule. Based on the above calculation, the electrochemically active surface area ($A$) of the WE was calculated to be 0.0212 ± 0.0019 cm^2^ (*n* = 4), and the immobilized aptamer density was determined to be (4.01 ± 1.51) × 10^11^ molecules/cm² (*n* = 4).

**Fluorescence verification of selective removal of SAM**

The selective removal of the SAM on WE2 was verified using SYBR Gold staining. The SYBR Gold stain was diluted 1:10000 in TE buffer (10 mM Tris-HCl, 1 mM EDTA, pH 8.0) to prepare a 1× staining solution. The IDE chip surface was incubated with the staining solution for 40 min at room temperature, protected from light. Fluorescence images were then acquired using an upright microscope (DM2700, Leica, Germany) equipped with an LED light source (pE-4000, CoolLED, UK) set to an excitation wavelength of 490 nm. Images were captured through the microscope’s eyepiece using a digital camera (EOS R5, Canon, Japan) attached *via* a camera adapter (TUST30S, LMscope, Austria). Camera settings were optimized to avoid signal saturation.

**AFM verification of functionalization of the IDE chip**

Atomic force microscopy (AFM) was performed using a Dimension V AFM system (Bruker/Veeco, Billerica, MA, USA) operated in tapping mode. Super sharp silicon probes (tip radius <2 nm, resonance frequency ~150 kHz) were employed for high-resolution surface imaging. All scans were conducted over a 5 μm × 5 μm or 3 μm × 3μm area at a scan rate of 1 Hz. Functionalized IDE chips were thoroughly rinsed with Milli-Q water and dried under a gentle nitrogen stream prior to imaging. Representative regions on each electrode were imaged to monitor topographical changes during surface functionalization steps, including bare Au, SAM formation, and SAM removal. Image analysis and surface roughness quantification were carried out using Nanoscope Analysis and WSxM software.^[5]^

**Electrochemical characterization of the biosensing platform**

All electrochemical measurements, including CV and square wave voltammetry (SWV) were performed using a potentiostat (PalmSens4, PalmSens BV, Netherlands) in a three-electrode configuration with an Ag/AgCl reference electrode, a Pt counter electrode, and an IDE chip as the working electrode. The CVs were conducted over a potential range of −0.1 V to 0.6 V at a scan rate of 100 mV/s in PBS containing 5 mM [Fe(CN)₆]⁴⁻/[Fe(CN)₆]³⁻ (1:1) and 0.1 M KCl. SWV was carried out from −0.5 V to 0 V with a frequency of 50 Hz. The SWV peak currents were extracted using PSTrace software.

**Numerical simulation**

Numerical simulations were performed using COMSOL Multiphysics 6.3 to investigate the the influence of an applied electric field on DNA transport between five pairs of spatially separated working electrodes (WE1 and WE2), as well as the effect of inter-electrode spacing. The system was modelled as a two-dimensional cross-section of an interdigitated electrode geometry. The system was modelled as a two-dimensional cross-section of an interdigitated electrode geometry, with the working electrodes at the bottom and a counter electrode at the top of the domain. As the geometry is symmetric, only one half of the IDE domain was simulated and a symmetry boundary condition added to one side of the model. A rectangular domain was defined to represent the bulk electrolyte solution, while thin rectangular domains were introduced above each working electrode to approximate the functionalized DNA layer and enable local mesh refinement. An out-of-plane thickness of 4 mm was used to mimic the fabricated device geometry. Key simulation parameters include the half-domain width (1400–3400 µm), domain height (1000 µm), electrode width (200 µm), effective DNA layer thickness (10 nm), DNA diffusion coefficient (1.18 × 10^−10^ m^2^ s^−1^), net DNA charge number (−11), initial DNA concentration (1 × 10^−3^ mol m^−3^), medium conductivity (1.79 S m^−1^), applied current on WE2 (41 nA), and the relative permittivity of water (εᵣ = 78.5).

The simulations coupled the COMSOL Electric Currents and Transport of Diluted Species interfaces. Mass transport of the DNA was described using the Nernst–Planck equation, while the electric field distribution in the electrolyte was obtained by solving for current conservation in the medium between WE2 and the counter electrode. The system was assumed to be chemically inert and isothermal, and convective transport was neglected. WE1 was left floating, while a current of 41 nA was applied between WE2 and the counter electrode to induce electrophoretic transport of negatively charged DNA.

Mesh refinement was applied in the thin domains above the WEs using a mapped mesh to capture concentration gradients along the electrode width, while a physics-controlled triangular mesh was used elsewhere. A mesh convergence study was performed to ensure solution independence. The total electric energy in the 2D system, as well as the electric field norm at the tips of WE2, were used as convergence criteria (<2% difference in results compared to a coarser mesh). The final mesh consisted of approximately 2.5 × 10^5^ elements.

**Biofluid sample detection**

To assess sensor performance in biological media, dopamine detection was first carried out in aCSF. Dopamine-spiked aCSF solutions at varying concentrations were prepared, and 30 μL of each solution was applied to the IDE chip to fully cover both WE1 and WE2. The chip was incubated at room temperature for 2 h, followed by rinsing with deionized water. Subsequent SWV measurements were conducted in fresh aCSF. Calibration curves were plotted based on the concentration-dependent signal changes at WE1 and WE2. For WE1, which exhibited a signal-off response, the linear range was fitted using the regression equation:

$$I=-\frac{2.63\mathrm{nA}}{\mathrm{nM}}\times C+503 \mathrm{nA} (R^{2}=0.889)$$

For WE2, which showed a signal-on response, the regression fit was:

$$I=\frac{1.36nA}{nM}\times C+75.9 \mathrm{nA} (R^{2}=0.998)$$

where *I* is the SWV peak current (nA) and *C* is the target concentration (nM).

The limit of detection (LOD) for each WE was calculated using the standard 3σ criterion:

$${LOD}_{WE1}=\mu_{WE1 control}-3\sigma_{WE1 control}$$

$${LOD}_{WE2}=\mu_{WE2 control}+3\sigma_{WE2 control}$$

Where $\mu_{control}$ represents the mean signal obtained from the control (blank) samples, and $\sigma_{control}$ represents the standard deviation of these measurements.

To accelerate the assay, an electric field-assisted diffusion strategy was employed. A bias potential of +0.5 V was applied to WE2 for 1 min prior to target incubation, while all other assay conditions remained unchanged.

For cortisol detection, both saliva and serum samples were analyzed. Serum samples were diluted to 50% (v/v) with 1×PBS prior to measurement. Saliva samples were collected at 9:00 p.m. from the first author using Salivette® cortisol collection tubes (Sarstedt, Nümbrecht, Germany). The swab was placed in the mouth for 2 min, transferred into the collection tube, and centrifuged at 1000 × g for 3 min. The collected saliva sample solution was then stored at −20 °C and diluted to 30% (v/v) with 1×PBS prior to use. Both diluted serum and saliva samples were spiked with varying concentrations of cortisol for subsequent analysis. The detection protocol followed the same procedures as previously described, except that electric field-assisted diffusion was applied on WE2 and the incubation time was reduced to 30 min.

**
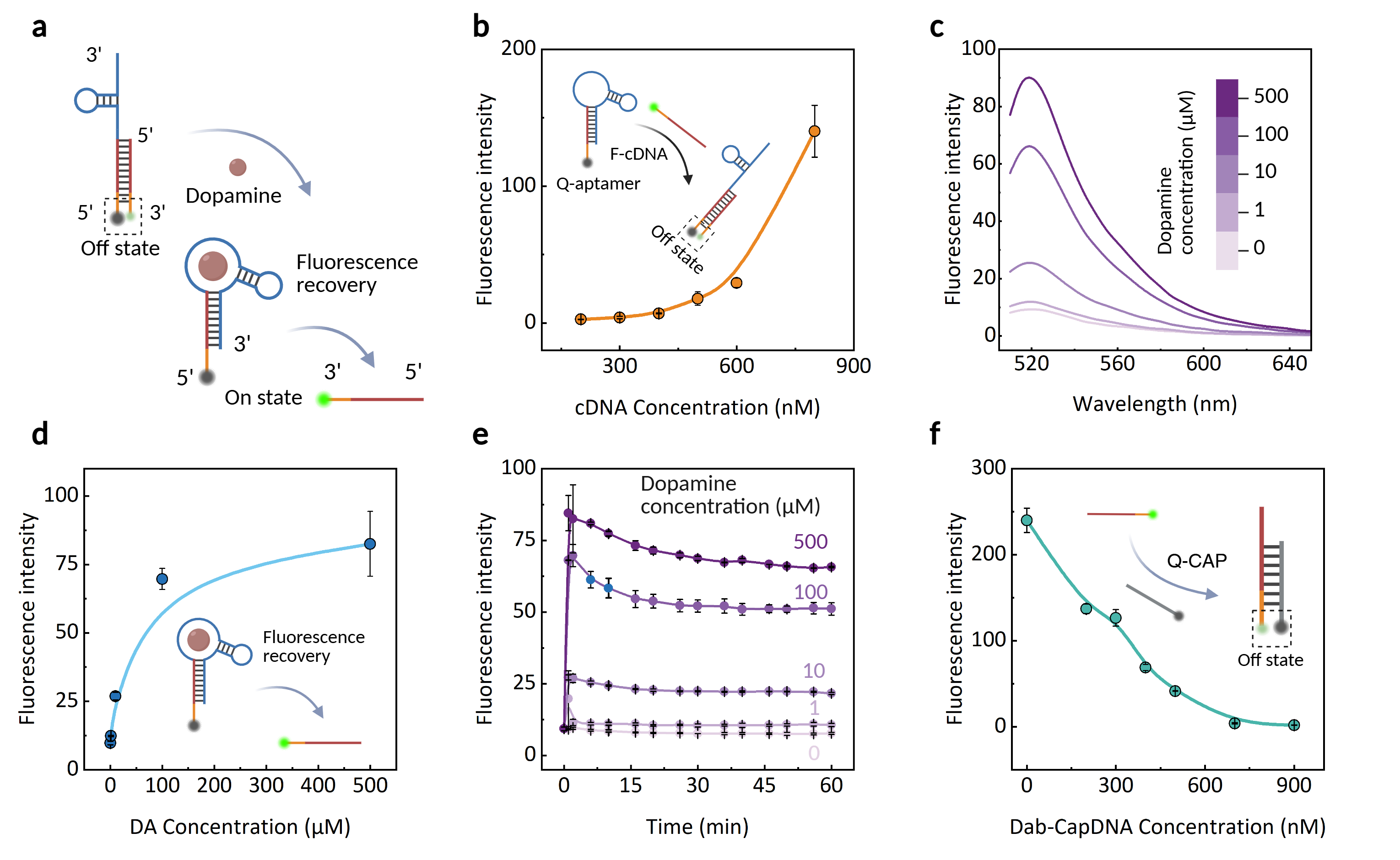
**

**Figure S1.** Fluorescence-based assay for characterization of the dopamine-responsive aptamer–cDNA duplex (using cDNA 14-5) and evaluation of capture probe recapture efficiency. (a) Schematic of the fluorescence activation mechanism. In the absence of dopamine, the F-cDNA remains quenched in the Q-aptamer duplex (off state). Upon dopamine binding, the aptamer undergoes a conformational change, releasing the quenched strand and restoring fluorescence. (b) Optimization of F-cDNA concentration. With fixed Q-aptamer levels (500 nM), fluorescence remained low when F-cDNA was below a 1:1 ratio due to full quenching. Excess F-cDNA led to increased fluorescence, indicating that a ~1:1 ratio is optimal for duplex formation. (c) Fluorescence emission spectra at increasing dopamine concentrations (0–500 μM), showing a concentration-dependent recovery of fluorescence. (d) Concentration–response calibration curve of fluorescence intensity versus dopamine concentration after 2 min of incubation. (e) Time-resolved fluorescence kinetics measured at various dopamine concentrations, indicating rapid signal recovery within the first 5–10 min. (f) Quenching assay for evaluating capture probe performance. Increasing concentrations of quencher-labeled capture DNA (Q-CAP) led to progressive fluorescence quenching of released F-cDNA, confirming effective hybridization and suitability for downstream recapture. Curves in (b), (d), (e), and (f) were fitted using B-spline plot for visual guidance. Data are presented as mean ± s.d. (*n* = 3).

**
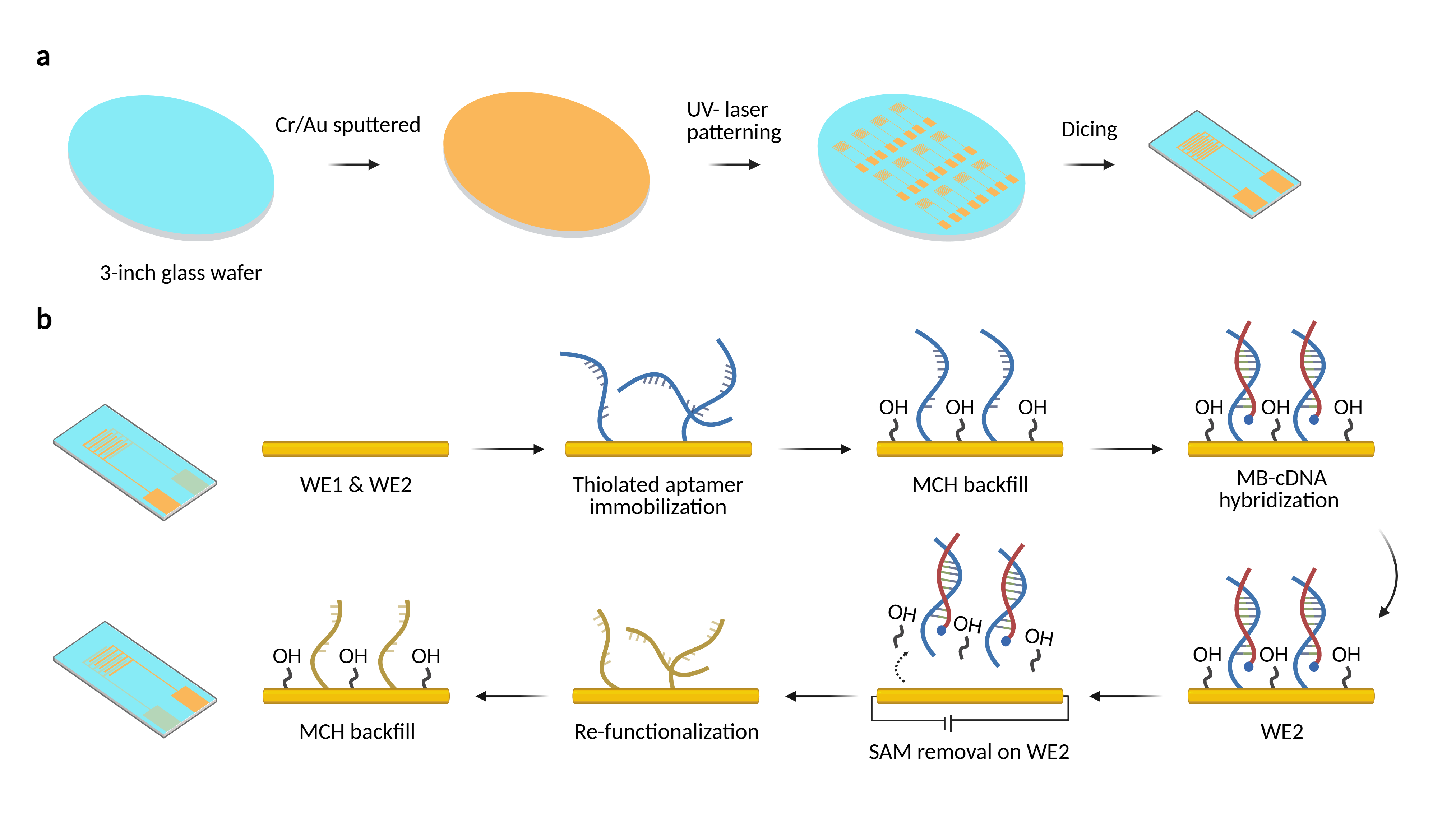
**

**Figure S2.** Schematic illustration of interdigitated electrode (IDE) chip fabrication and surface functionalization. (a) Fabrication process of IDE chips: A 3-inch glass wafer is coated with a Cr/Au layer *via* sputtering, followed by UV laser patterning and dicing into individual sensor chips. (b) Surface modification steps on the IDE chip. Both working electrodes (WE1 and WE2) are initially modified with thiolated aptamers and backfilled with 6-mercapto-1-hexanol (MCH), followed by hybridization with methylene blue-labeled complementary DNA (MB-cDNA). Subsequently, the SAM on WE2 is selectively removed and re-functionalized with a capture probe (CAP) to enable dual-channel signal output. The MCH is reapplied to block nonspecific adsorption on WE2.

**
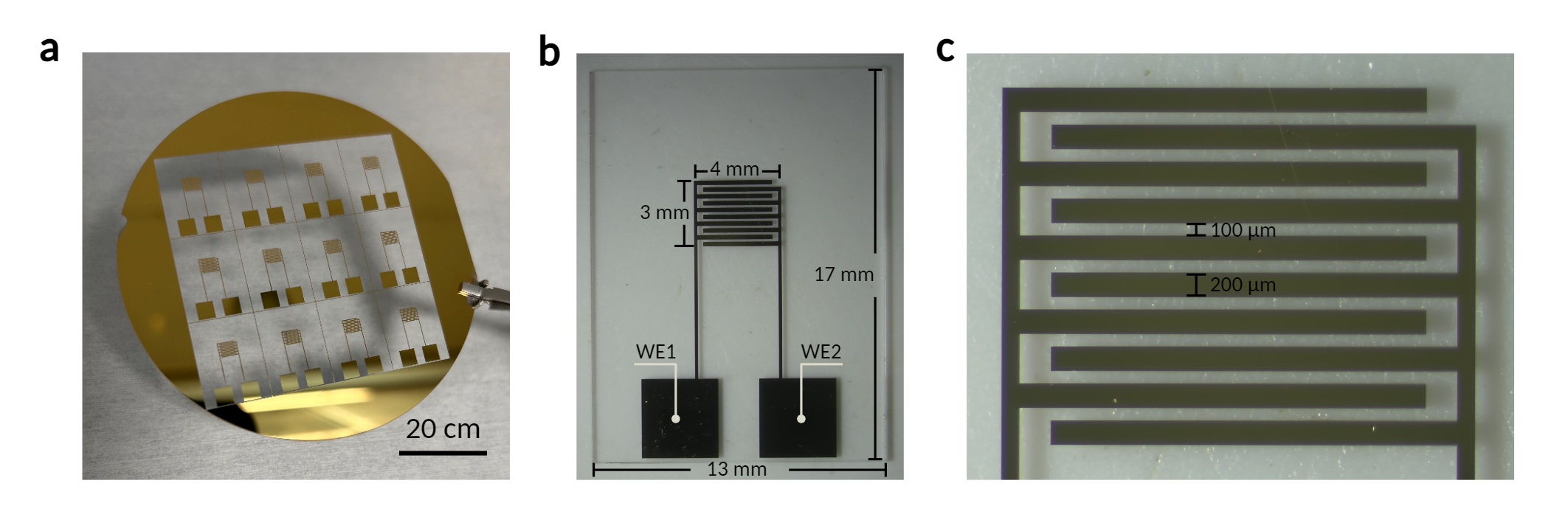
**

**Figure S3.** Photographic and microscopic images of the fabricated IDE chip. (a) Photograph of a 3-inch Cr/Au-coated glass wafer after UV laser patterning, showing 12 chips layouts prior to dicing. (b) Optical image of a single diced IDE chip with two working electrodes (WE1 and WE2). Dimensions of the electrode layout are indicated. (c) Close-up image of the interdigitated region, showing parallel electrode bands with 200 µm width and 100 µm spacing.

**
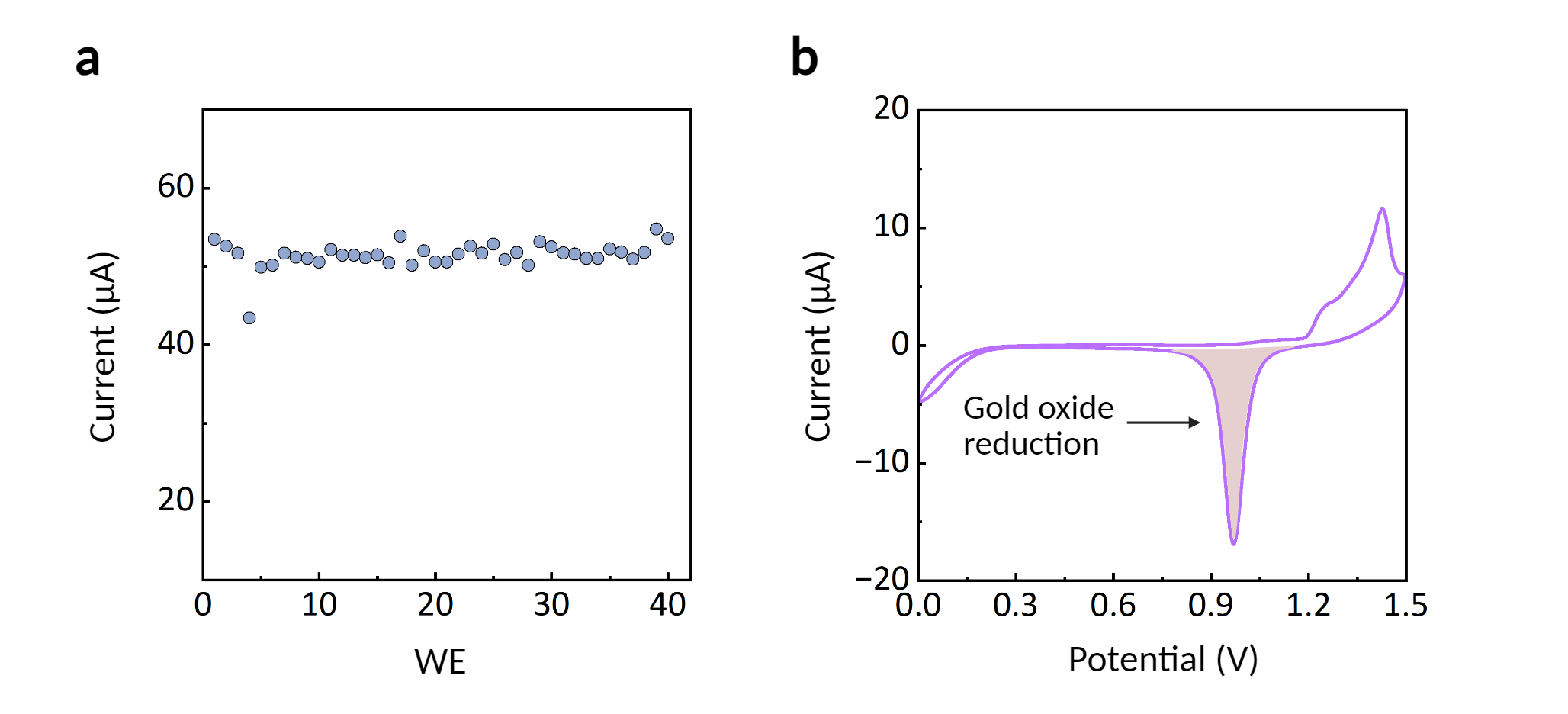
**

**Figure S4.** Electrochemical characterization and reproducibility of Au working electrodes. (a) Inter-electrode variability assessed by measuring the CV reduction peak current of 40 independently fabricated WEs, demonstrating high fabrication uniformity. (b) CV of a representative gold electrode in 0.1 M H₂SO₄, showing a distinct gold oxide reduction peak at approximately 1.0 V.

**
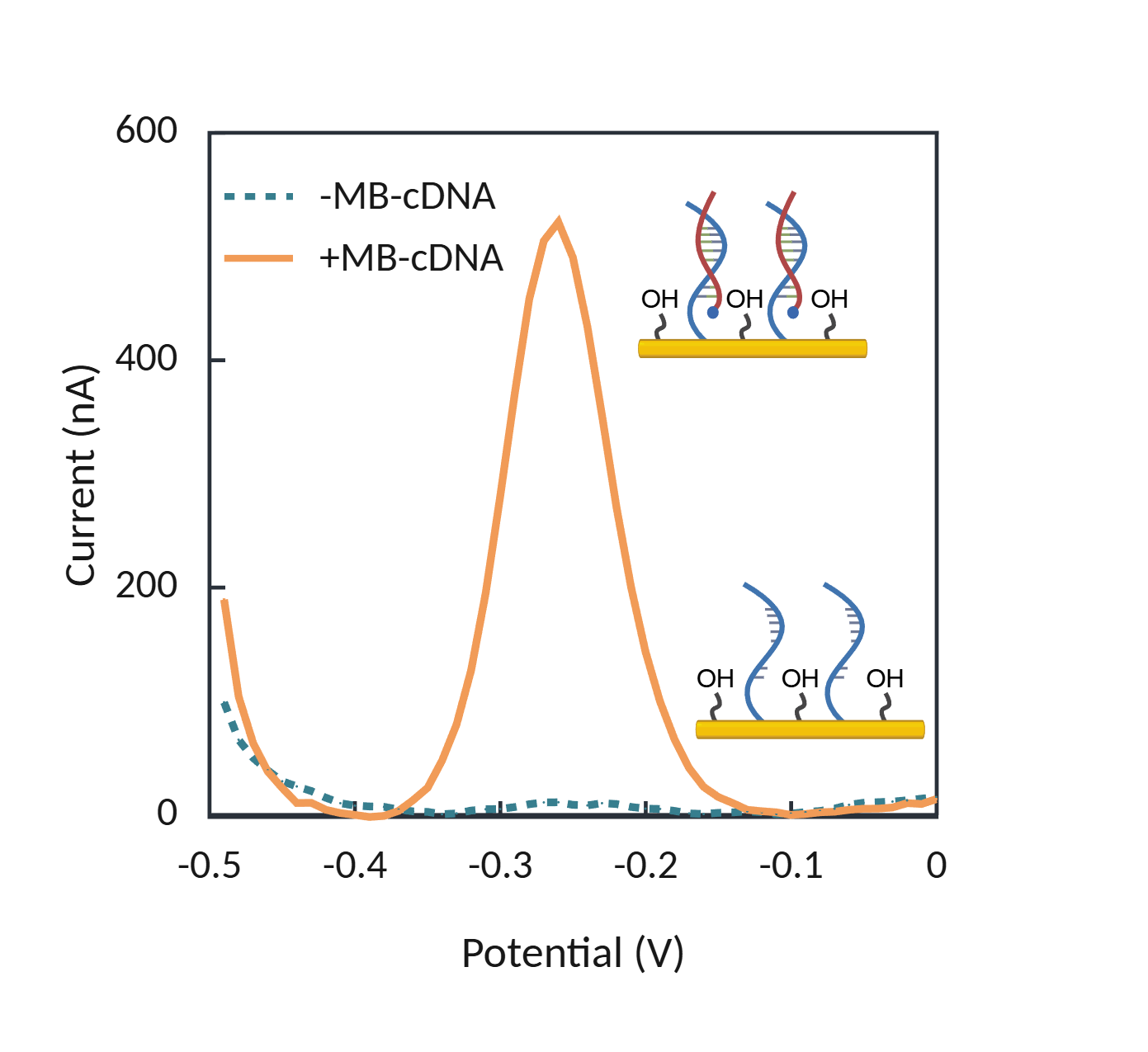
**

**Figure S5.** Square wave voltammetry (SWV) response before and after hybridization with MB-cDNA on the electrode surface. A typical redox peak near −0.28 V appears upon hybridization of MB-cDNA with surface-tethered aptamers, confirming successful duplex formation and indicating effective probe immobilization.


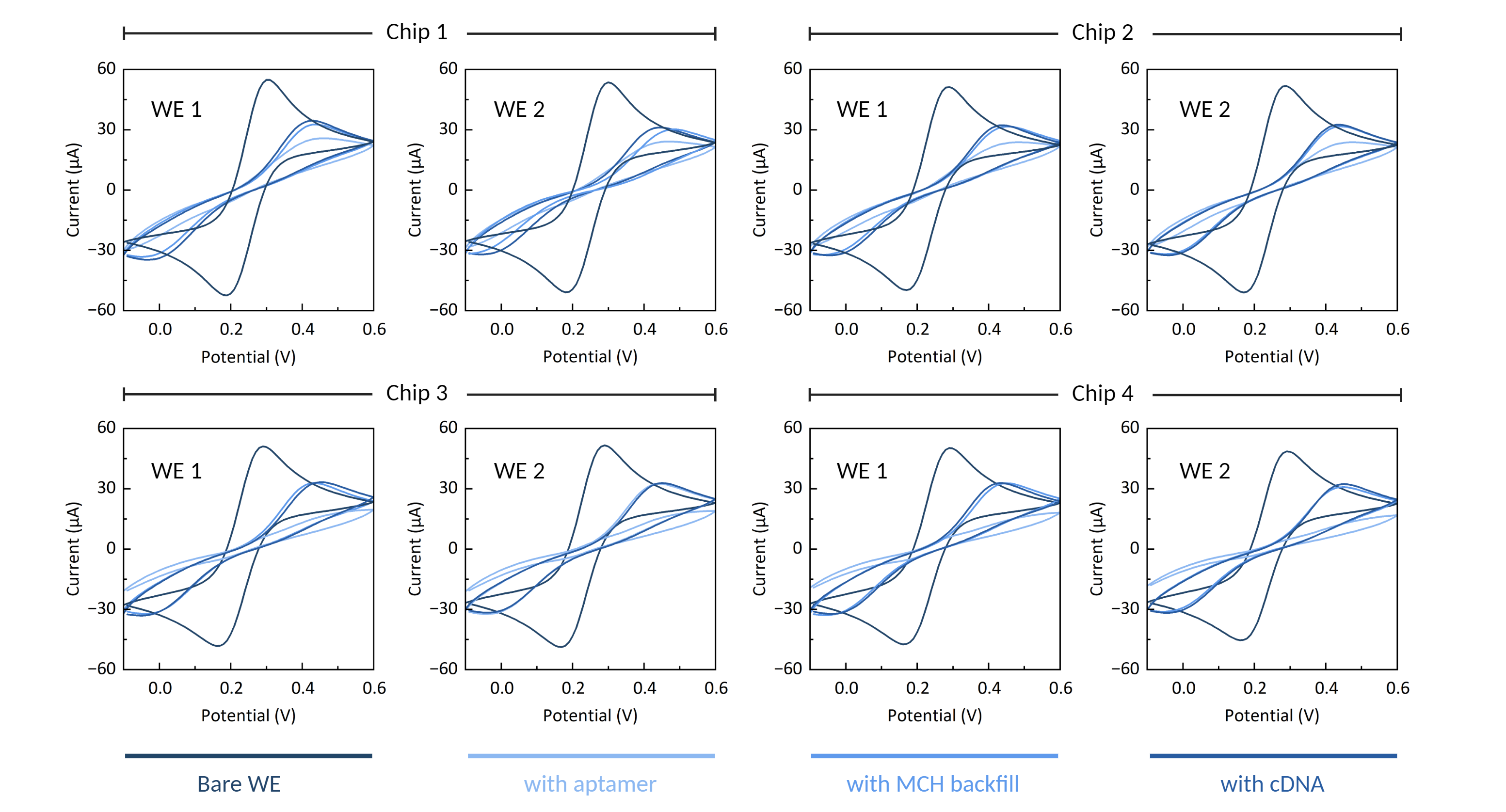


**Figure S6.** Reproducibility of WE surface functionalization evaluated by CV. The CV from four representative chips (Chip 1–4) show consistent current responses across WE1 and WE2 at each functionalization step: bare gold, aptamer immobilization, MCH backfilling, and cDNA hybridization. Similar profiles across chips demonstrate reproducibility of the functionalization process.

**
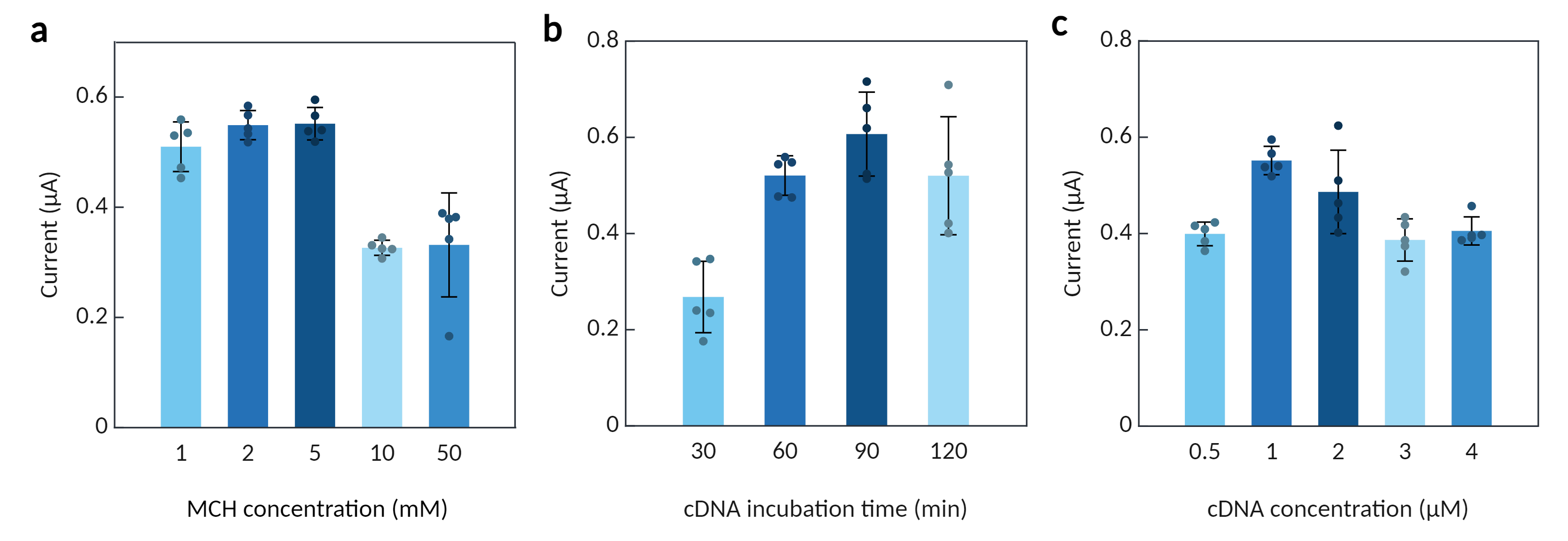
**

**Figure S7.** Optimization of the surface modification parameters. (a) Effect of MCH concentration on current after aptamer immobilization and MB-cDNA hybridization. Moderate concentrations (2–5 mM) yielded higher signals, while excessive MCH reduced current due to over passivation of the electrode surface. (b) Influence of MB-cDNA incubation time on signal output. Current increased with longer incubation and plateaued around 90 min, suggesting completion of surface hybridization. (c) Effect of MB-cDNA concentration on current response. A maximum signal was observed at 1 µM, beyond which additional MB-cDNA had no beneficial effect, indicating saturation of surface-bound aptamers. All measurements were performed using SWV. Data are shown as mean ± s.d. (*n*= 5).

**
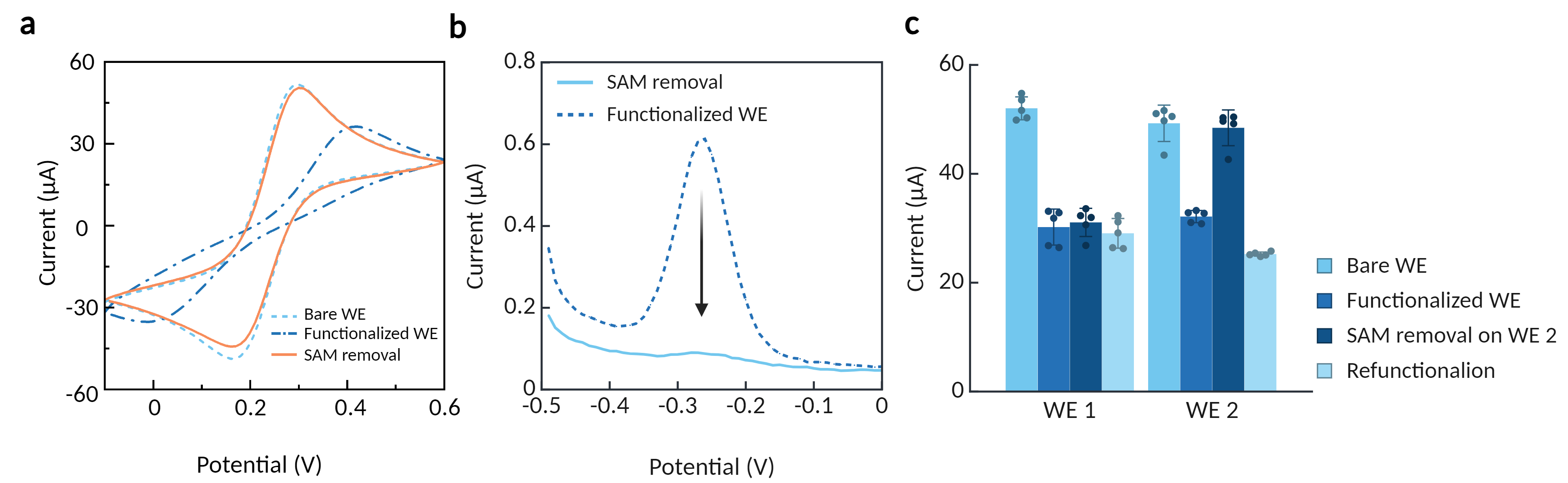
**

**Figure S8.** Electrochemical validation of self-assembled monolayer (SAM) removal and electrode re-functionalization. (a) Cyclic voltammograms (CVs) of the same electrode at different stages. The SAM removal restores the redox current toward that of the bare electrode, indicating successful re-exposure of the gold surface. (b) SWV signal before and after SAM removal. The MB-cDNA redox peak (approximately −0.28 V) disappears following SAM stripping, confirming effective removal of the duplex layer. (c) Summary of peak currents at WE1 and WE2 across functionalization stages: bare electrode, aptamer functionalization, SAM removal on WE2, and re-functionalization with CAP. Data are presented as mean ± s.d. (*n* = 5). SAM removal was performed by applying a potential of −1.1 V for 250 s.

**
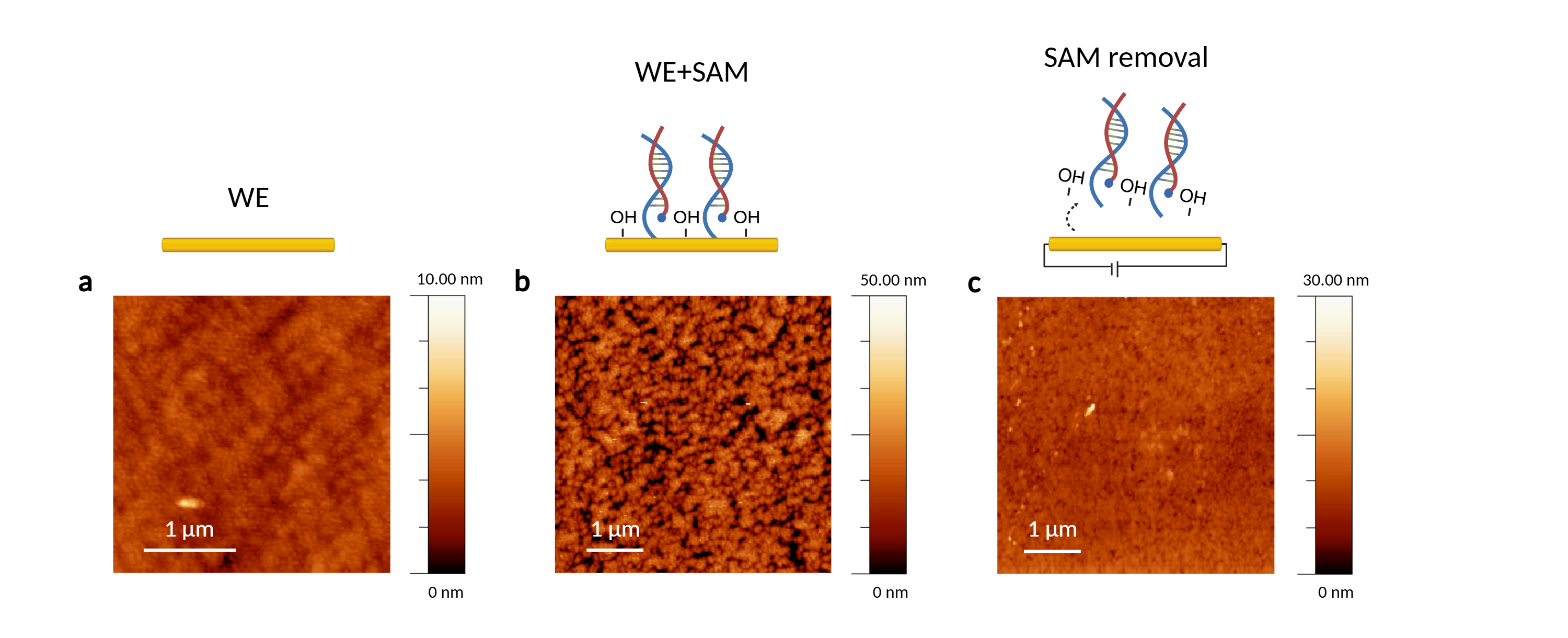
**

**Figure S9.** Atomic force microscopy **(**AFM) characterization of surface functionalization and SAM removal on IDE chips. (a) Bare WE showing a relatively smooth surface (mean roughness: 0.643 ± 0.075 nm). (b) WE after SAM formation exhibits increased surface roughness (5.04 ± 0.44 nm), indicating successful molecular assembly. (c) WE following electrochemical SAM removal (−1.1V for 250 s) shows a substantial decrease in surface features and roughness (1.39 ± 0.40 nm), similar to the initial bare surface.


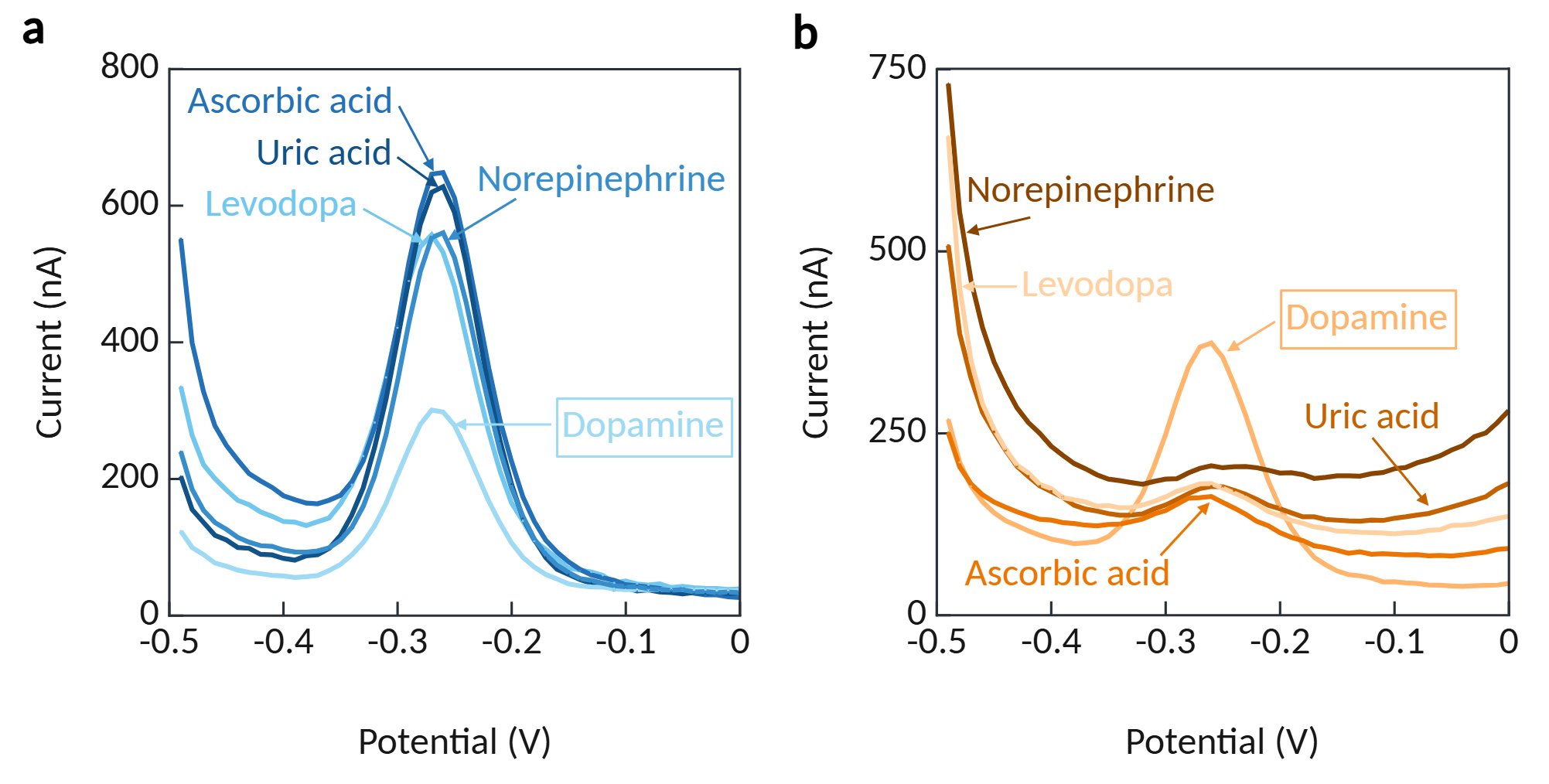


**Figure S10.** Specificity of the dual-channel IDE aptamer-modified sensing chip against structurally related or electroactive interferents. (a) SWV responses at WE1 in the presence of 100 nM of various potentially interfering substances, including ascorbic acid, uric acid, norepinephrine, and levodopa. Only dopamine induced a significant current decrease at the characteristic redox potential (approximately −0.28 V). (b) Corresponding SWV responses at WE2 under the same conditions. Dopamine yielded a distinct signal, while other interferents produced negligible changes, confirming the specificity of both recognition and recapture processes across the dual-channel platform.

**
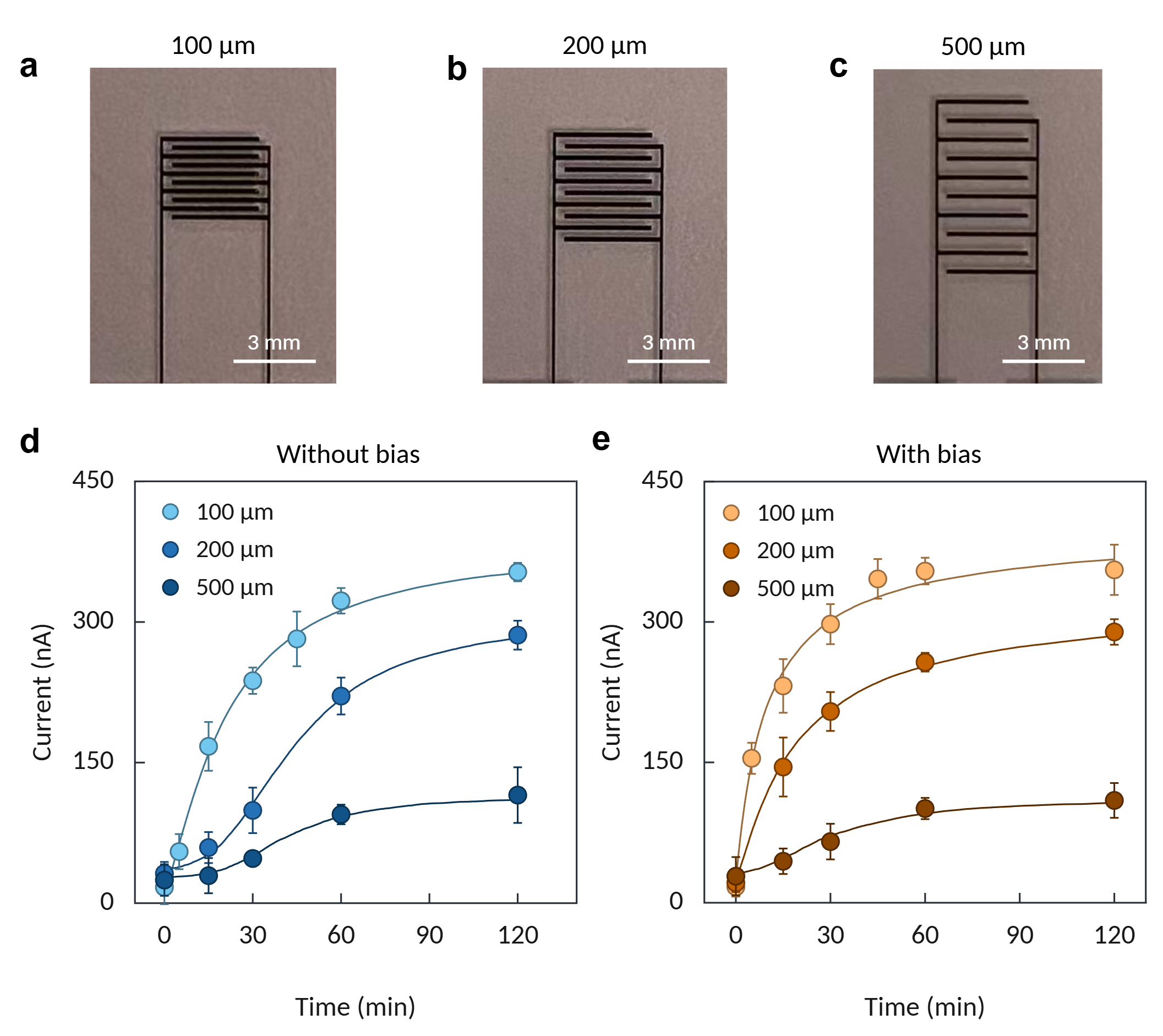
 Figure S11.** Characterization of IDE chips with varying inter-electrode spacing and its impact on signal probe transport. (a-c) Photograph of the IDE chips fabricated on glass substrate with 100, 200, 500 µm inter-electrode gaps respectively. (d, e) Time-resolved current responses measured at WE2, reflecting the migration of the released MB-cDNA probe across IDEs with different spacings over a 120 min period, recorded (d) without and (e) with bias-assisted transport. Data are presented as mean ± s.d. from three independent measurements (*n* = 3).

**
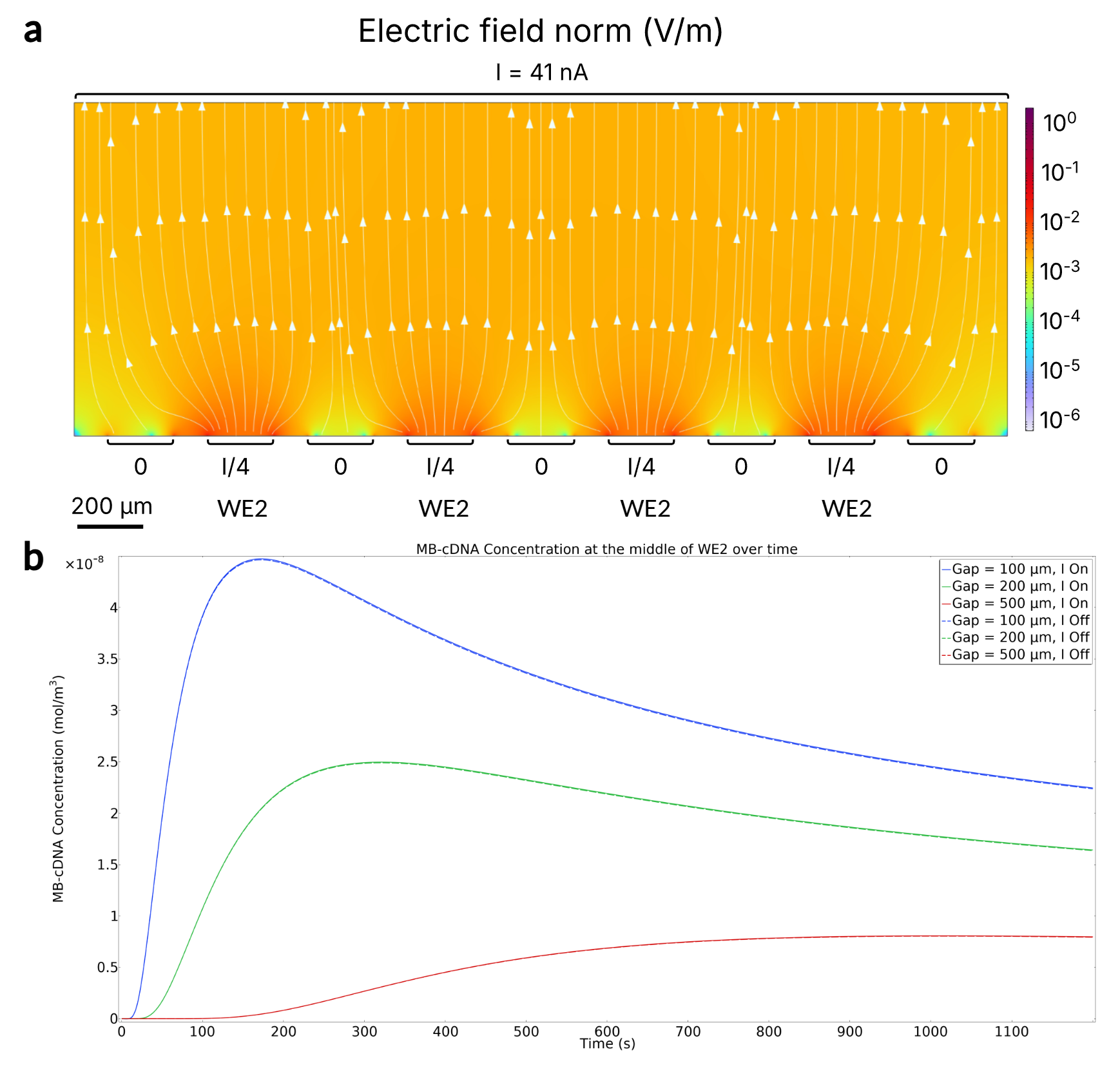
**

**Figure S12.** Numerical simulation of bias-assisted DNA transport between interdigitated working electrodes (WEs). (a) Simulated electric field norm and field lines with a background current of 41 nA, corresponding to an applied +0.5 V bias in the experiment. The simulation shows field localization at the electrode edges. (b) Time-dependent MB-cDNA concentration at the center of WE2 for different inter-electrode gaps (100, 200, and 500 µm), with (solid lines) and without (dashed lines) a background current on WE2. While reducing the inter-electrode spacing markedly accelerates probe accumulation at WE2, the applied background current produces only a minor effect on MB-cDNA transport under the simulated conditions. These results suggest that electrophoretic migration alone is insufficient to account for the experimentally observed bias-assisted enhancement, indicating that additional mechanisms (such as electroosmosis) likely contribute to the accelerated signal transduction.

**
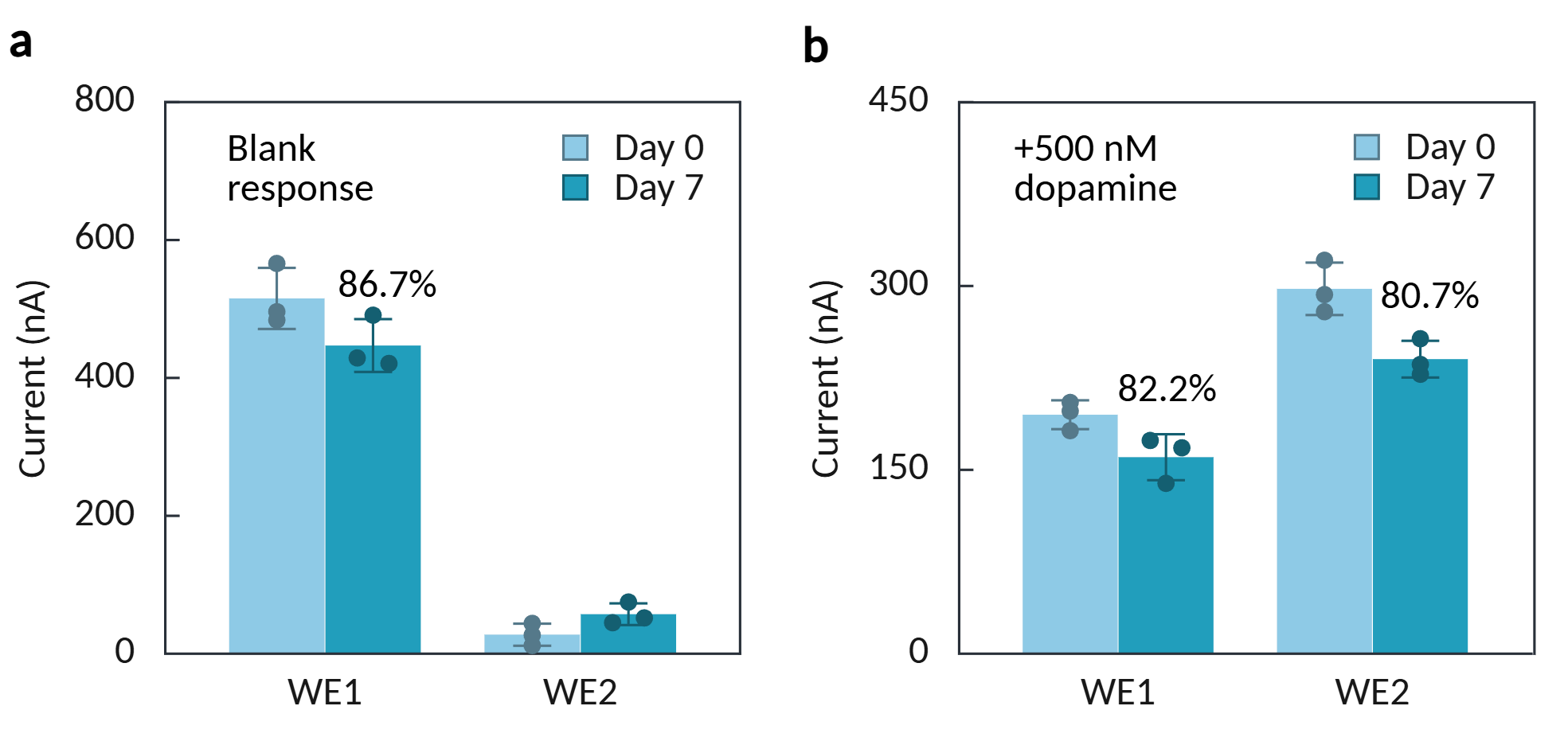
**

**Figure S13.** Long-term storage stability of the fully functionalized dual-channel IDE aptamer-modified sensing chips. The chips were stored in PBS at 4 °C and characterized on Day 0 and after 7 days. (a) Blank SWV responses measured at WE1 and WE2 in the absence of target. After 7 days of storage, WE1 retained 86.7% of its initial signal, while a slight signal increase was observed at WE2, likely due to nonspecific release and recapture of MB-cDNA from WE1. (b) Sensor responses at WE1 and WE2 upon exposure to 500 nM dopamine. After 7 days of storage, WE1 and WE2 retained 82.2% and 80.7% of their initial signals, respectively. All measurements were performed using SWV and measured using the electric bias-assisted diffusion strategy followed by a 30-min incubation. Data are presented as mean ± s.d. (*n* = 3).

**
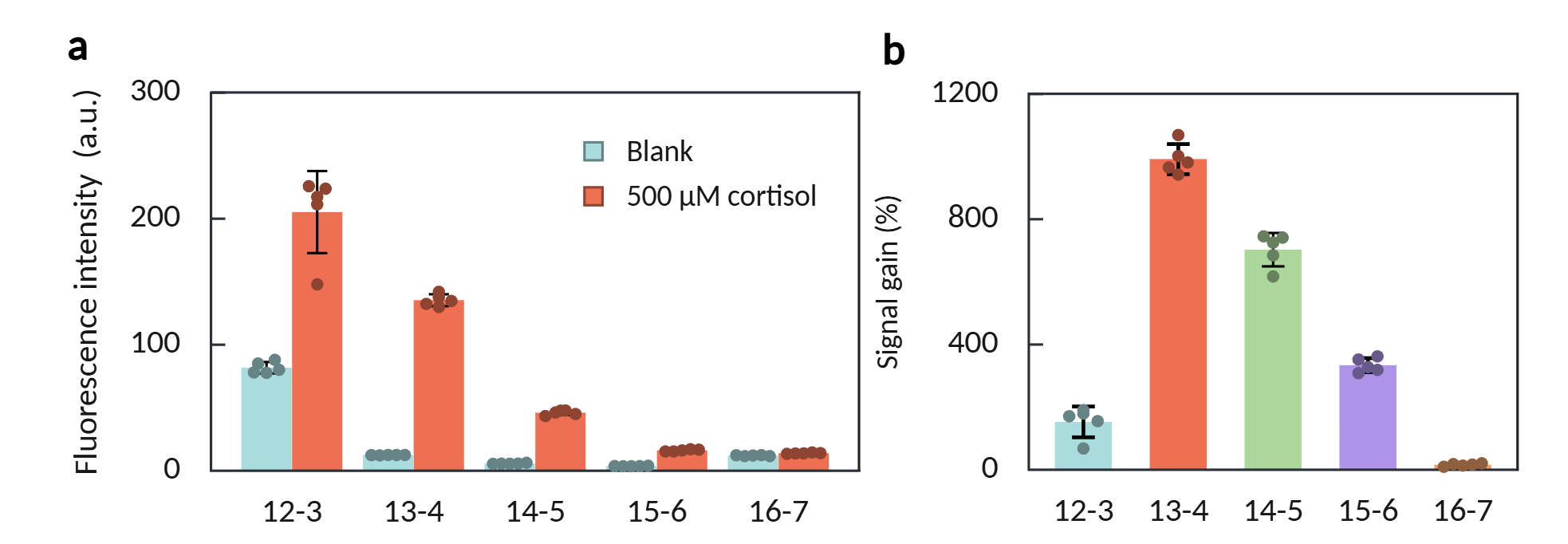
**

**Figure S14.** Screening of cDNA sequences for optimal hybridization with the cortisol aptamer. (a) Fluorescence intensity of candidate FAM-labeled cDNA sequences (12-3, 13-4, 14-5, 15-6, 16-7) hybridized with the cortisol aptamer in the absence (blank) and presence of cortisol (500 µM). (b) Corresponding signal gain calculated from (a), candidate13-4 exhibited the highest signal gain, indicating the most efficient target-induced displacement behavior. Aptamer and cDNA concentrations were 500 nM; Data are presented as mean ± s.d. (*n* = 5).

**
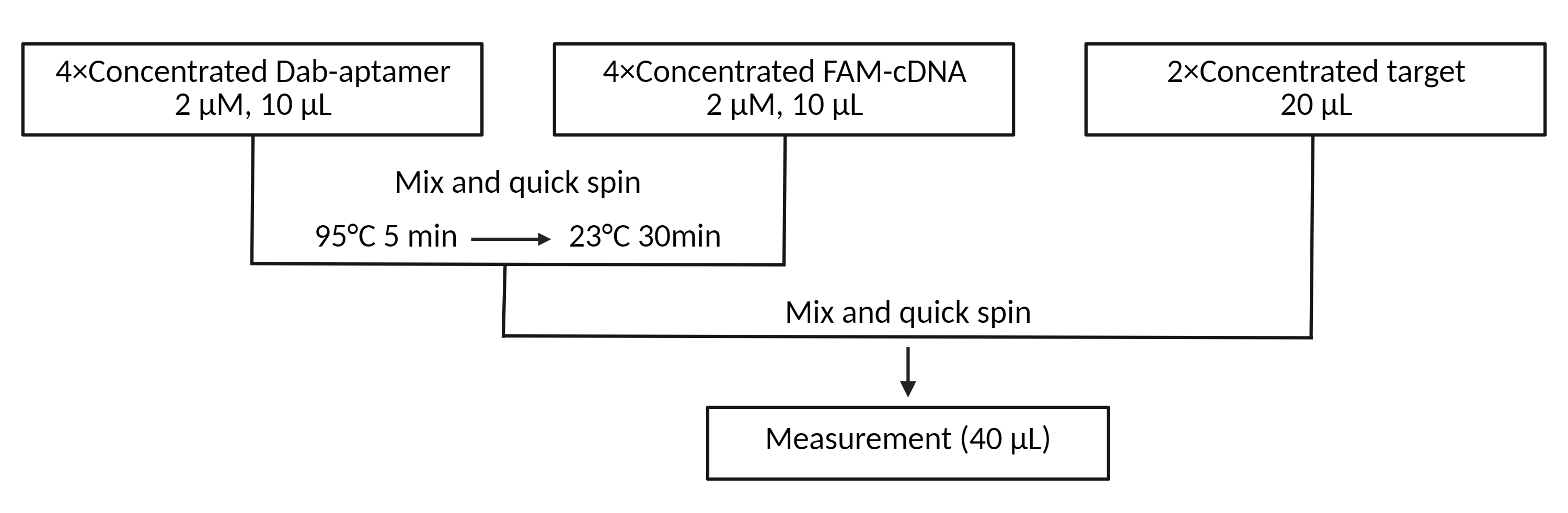
**

**Figure S15.** Fluorescence assay protocol for evaluating aptamer-cDNA displacement. Equal volumes of 4× concentrated Dab-aptamer (2 µM, 10 µL) and 4× concentrated FAM-labeled cDNA (2 µM, 10 µL) were mixed, briefly vortexed, and annealed by heating at 95 °C for 5 min followed by cooling to 23 °C for 30 min. Subsequently, 2× concentrated target (20 µL) was added to the duplex solution, mixed, and briefly vortexed. The final 40 µL mixture was used for fluorescence measurement.

**Table S1. Fluorescence response of dopamine aptamer with different cDNA sequences**

| cDNA candidates | Fluorescence intensity (a.u.) | | Signal gain (%) | SD |
| --- | --- | --- | --- | --- |
|  | Blank | +500 μM dopamine |  |  |
| 12-3 | 43.2 | 82.4 | 90.8 | 8.7 |
|  | 47.8 | 87.4 | 82.7 |  |
|  | 47.6 | 82.6 | 73.4 |  |
| 13-4 | 20.2 | 88.2 | 335.9 | 28.3 |
|  | 21.2 | 80.9 | 282.1 |  |
|  | 22.6 | 89.1 | 294.0 |  |
| 14-5 | 7.0 | 69.5 | 893.0 | 159.2 |
|  | 6.9 | 83.8 | 1112.3 |  |
|  | 7.1 | 64.5 | 802.6 |  |
| 15-6 | 4.5 | 21.1 | 370.2 | 38.1 |
|  | 5.0 | 19.6 | 295.2 |  |
|  | 4.7 | 20.0 | 320.9 |  |
| 16-7 | 3.3 | 7.9 | 137.9 | 9.2 |
|  | 3.6 | 8.0 | 119.6 |  |
|  | 3.4 | 7.8 | 131.0 |  |

SD: standard deviation of signal gain.

**Table S2. Fluorescence response of cortisol aptamer with different cDNA sequences**

| cDNA candidates | Fluorescence intensity (au.) | | Signal gain (%) | SD |
| --- | --- | --- | --- | --- |
|  | Blank | + 500μM cortisol |  |  |
| 12-3 | 78.0 | 211.4 | 171.0 | 49.1 |
|  | 77.7 | 225.8 | 190.5 |  |
|  | 80.1 | 223.8 | 179.5 |  |
|  | 85.1 | 217.3 | 155.3 |  |
|  | 88.0 | 147.9 | 68.1 |  |
| 13-4 | 12.4 | 132.4 | 965.2 | 48.1 |
|  | 12.2 | 142.0 | 1068.7 |  |
|  | 12.5 | 129.9 | 942.5 |  |
|  | 12.5 | 137.6 | 1001.7 |  |
|  | 12.5 | 134.7 | 981.1 |  |
| 14-5 | 5.5 | 43.4 | 684.8 | 53.4 |
|  | 5.6 | 47.7 | 745.3 |  |
|  | 5.6 | 46.2 | 725.3 |  |
|  | 5.7 | 47.8 | 741.2 |  |
|  | 6.3 | 45.0 | 617.3 |  |
| 15-6 | 3.7 | 15.3 | 308.6 | 22.5 |
|  | 3.6 | 15.3 | 327.7 |  |
|  | 3.6 | 16.1 | 352.1 |  |
|  | 3.7 | 17.1 | 362.1 |  |
|  | 4.0 | 16.7 | 319.0 |  |
| 16-7 | 12.3 | 13.4 | 9.6 | 4.3 |
|  | 11.6 | 13.7 | 18.2 |  |
|  | 12.1 | 13.8 | 13.5 |  |
|  | 12.4 | 14.5 | 16.7 |  |
|  | 11.6 | 14.0 | 20.7 |  |

SD: standard deviation of signal gain.

**Table S3.** **DNA sequences used in this study.**

| DNA used in the fluorescence assay | |
| --- | --- |
| Name | Sequences (5'-3') |
| Dopamine-aptamer | /5Dabcyl/GAGAGGGGACGACGCCAGTTTGAAGGTTCGTTCGCAGGTGTGGAGTGACGTCGTCCC |
| Cortisol-aptamer | /5Dabcyl/GAGAGGGGACGACGCCCGCATGTTCCATGGATAGTCTTGACTAGTCGTCCC |
| cDNA 12-3 | TCGTCCCCTCTC/36-FAM/ |
| cDNA 13-4 | GTCGTCCCCTCTC/36-FAM/ |
| cDNA 14-5 | CGTCGTCCCCTCTC/36-FAM/ |
| cDNA 15-6 | GCGTCGTCCCCTCTC/36-FAM/ |
| cDNA 16-7 | GGCGTCGTCCCCTCTC/36-FAM/ |
| CAP 9 | GAGAGGGGA |
| CAP 11 | GAGAGGGGACG |
| CAP 13 | GAGAGGGGACGAC |
| CAP 14 | GAGAGGGGACGACG |
| Dab-CAP 9 | /5Dabcyl/GAGAGGGGA |
| DNA used in the electrochemical assay | |
| Name | Sequences (5'-3') |
| Dopamine-aptamer | /5ThiolC6/GAGAGGGGACGACGCCAGTTTGAAGGTTCGTTCGCAGGTGTGGAGTGAC GTCGTCCC |
| Cortisol-aptamer | /5ThiolC6/GAGAGGGGACGACGCCCGCATGTTCCATGGATAGTCTTGACTAGTCGTCCC |
| MB-cDNA  (dopamine assay) | CGTCGTCCCCTCTC/3Methylene blue/ |
| MB-cDNA  (cortisol assay) | GTCGTCCCCTCTC/3Methylene blue/ |

The dopamine aptamer and cDNA sequences were adapted with minor modifications from previous published work.^[6]^ and the cortisol aptamer sequence from Yang et al. (2017).^[7]^ /5Dabcyl/ indicates a 5' end Dactyl quencher modification. /36-Fam/ indicates a 3' end 6-carboxyfluorescein (6-FAM) fluorophore modification. /5ThiolC6/ indicates a 5' thiol modification with a 6-carbon linker. /3Methylene blue/ indicates a 3' end methylene blue label.

**Table S4.** **Compositions of the buffer and solutions used in this study**.

| Salt | Concentration (mM) | | |
| --- | --- | --- | --- |
|  | PBS containing Mg^2+^ and Ca^2+^ | PBS containing 5 mM K₄[Fe(CN)₆]/K₃[Fe(CN)₆] | aCSF |
| NaCl | 137 | 137 | 147 |
| KCl | 2.68 | 100 | 3.5 |
| Na_2_HPO_4_ | 10 | 10 | - |
| KH_2_PO_4_ | 1.76 | 1.76 | - |
| MgCl_2_ | 2 | - | 1.2 |
| CaCl_2_ | 1 | - | 1 |
| K_3_[Fe(CN)_6_] | - | 5 | - |
| K_4_[Fe(CN)_6_] | - | 5 | - |
| NaH_2_PO_4_ | - | - | 1 |
| NaHCO_3_ | - | - | 2.5 |

All solutions were adjusted to pH 7.4 before use.

**References**

[1] L. Niu, W. Knoll, *Anal. Chem.* **2007**, 79, 2695.

[2] D. Sen, R. A. Lazenby, *Anal. Chem.* **2023**, 95, 6828.

[3] A. B. Steel, T. M. Herne, M. J. Tarlov, *Anal. Chem.* **1998**, 70, 4670.

[4] J. Zhang, S. Song, L. Wang, D. Pan, C. Fan, *Nat. Protoc.* **2007**, 2, 2888.

[5] I. Horcas, R. Fernández, J. M. Gómez-Rodríguez, J. Colchero, J. Gómez-Herrero, A. M. Baro, *Rev. Sci. Instrum.* **2007**, 78.

[6] N. Nakatsuka, K.-A. Yang, J. M. Abendroth, K. M. Cheung, X. Xu, H. Yang, C. Zhao, B. Zhu, Y. S. Rim, Y. Yang, P. S. Weiss, M. N. Stojanović, A. M. Andrews, *Science* **2018**, 362, 319.

[7] K.-A. Yang, H. Chun, Y. Zhang, S. Pecic, N. Nakatsuka, A. M. Andrews, T. S. Worgall, M. N. Stojanovic, *ACS Chem. Biol.* **2017**, 12, 3103.
